# Rumen-Degradable Starch Improves Rumen Fermentation, Function, and Growth Performance by Altering Bacteria and Its Metabolome in Sheep Fed Alfalfa Hay or Silage

**DOI:** 10.3390/ani15010034

**Published:** 2024-12-26

**Authors:** Wenliang Guo, Meila Na, Shuwei Liu, Kenan Li, Haidong Du, Jing Zhang, Renhua Na

**Affiliations:** 1 College of Animal Science, Inner Mongolia Agricultural University, Hohhot 010018, China; 18686197338@163.com (W.G.);; 2 Grassland Research Institute of Chinese Academy of Agricultural Sciences, Hohhot 010010, China

**Keywords:** alfalfa, rumen-degradable starch, rumen fermentation, growth performance, urea transporter, bacteria and metabolome, microbial protein synthesis

## Abstract

Alfalfa silage due to its high protein can lead to easier feeding management, but its high proportion of rumen-degradable protein can reduce rumen nitrogen utilization. Nevertheless, increasing dietary energy can enhance ruminal microbial protein synthesis. Thirty-two Suffolk female sheep were used in this study, with a 2 × 2 factorial arrangement of treatment. The four treatments were a combination of two forage types (alfalfa hay; AH vs. alfalfa silage; AS) and two rumen-degradable starch levels (low RDS; LR vs. high RDS; HR) with a 15 d adaptation and 60 d experimental period. The rumen content and rumen epithelium samples were collected after slaughter. Feeding AS increased the rumen isobutyrate, valerate, ammonia-N (NH_3_-N) concentration, urase activity, and papillae height (*p* < 0.05) and reduced the feed to gain (F:G), rumen bacterial protein (BCP), rumen lactic acid concentration, and papillae width (*p* < 0.05) of sheep. Increased RDS in the diet improved the daily matter intake, average daily gain, and rumen weight, reduced the F:G, and enhanced the rumen nitrogen capture rate by decreasing total amino acids and the NH_3_-N concentration to increase BCP, aquaporins 3 gene, and protein expression. The rumen microbiota also changed as the HR diet reduced the Chao index (*p* < 0.05). The metabolomics analysis showed that feeding AS upregulated the rumen tryptophan metabolism and steroid hormone biosynthesis, while the purine metabolism, linoleic acid metabolism, and amino acid biosynthesis were downregulated. Furthermore, increased RDS in the diet upregulated rumen lysine degradation and sphingolipid metabolism, while aromatic amino acid biosynthesis was downregulated. Additionally, the correlation analysis results showed that ADG was positively correlated with 5-aminopentanoic acid, and three microorganisms (unclassified_f__Selenomonadaceae, Quinella, Christensenellaceae_R-7_group) were positively correlated with the rumen isobutyrate, valerate, NH_3_-N concentration, urase activity, tryptophan metabolism, and steroid hormone biosynthesis and negatively correlated with linoleic acid metabolism and amino acid biosynthesis in sheep. In summary, increased RDS in the diet improved the growth performance and rumen N utilization and reduced bacterial diversity in sheep. The alfalfa silage diet only increased feed efficiency; it did not affect growth performance. Additionally, it decreased rumen nitrogen utilization, linoleic acid, and amino acid biosynthesis. Nevertheless, there were limited interactions between forage and RDS; increased RDS in the AS diet enhanced the nitrogen capture rate of rumen microorganisms for alfalfa silage, with only slight improvements in the purine metabolism, linoleic acid, and amino acid synthesis.

## Simple Summary

The high rumen-degradable protein content of alfalfa silage (AS) limits its widespread application in ruminants. Increasing rumen-degradable starch (RDS) may enhance its rumen nitrogen efficiency. This study investigated the effects of alfalfa forage type (alfalfa hay; AH vs. alfalfa silage; AS) and RDS levels (low RDS vs. high RDS) on growth performance, rumen nitrogen efficiency, bacterial community, metabolome, and rumen function in sheep. The results showed that high RDS improved growth performance, rumen weight, and rumen bacterial nitrogen capture, as well as gene and protein expression of AQP3 in sheep. However, the interaction between alfalfa forage and RDS was limited. Although RDS improved nitrogen capture in AS, it only provided slight improvements in purine metabolism, linoleic acid metabolism, and amino acid synthesis.

## 1. Introduction

Alfalfa is a high-quality roughage because of its excellent protein and mineral content. Haymaking and ensiling are common practices for preserving alfalfa. However, losses in alfalfa hay (AH) during transportation and feeding operations have been estimated at 30%, making feed management more difficult [1]. Therefore, alfalfa silage (AS) has become a popular forage because of its complete nutritional preservation, and it can work in combination with concentrate wells to reduce dust [2,3]. However, its proportion of protein in the form of non-protein nitrogen (NPN) is higher due to proteolysis during preservation [4]. The ammonia–nitrogen (NH_3_-N) pool and non-ammonia NPN pool are substantially larger than in AH, and the proportion of rumen-degradable protein (RDP) is increased [5,6]. When ruminants are fed a greater proportion of AS, omasal total microbial protein flow and total N excretion increased, meaning that the efficiency of N utilization is lower [7,8,9], which has adverse economic and environmental implications.

Rumen microorganisms can synthesize microbial protein with a high efficiency using NPN, amino acid, and peptides, which are preferred N sources for ruminal microorganisms, and sufficient energy is critical for ruminal bacterial protein (BCP) synthesis. Some previous research results [7,10,11] suggest that enhancing the rate of ruminal fermentable energy may increase the microbial capture of NPN from AS, thereby reducing nitrogen excretion. However, the results were not consistent; the synchronization of rumen protein and carbohydrate was not achieved in practical production [12,13]. Ouellet et al. [14] found that the synchronization of rumen protein and energy had no effect on rumen bacterial protein and growth performance, but the energy infusion had more total and microbial N than the protein infusion [15]. Thus, synchronizing the ruminal availability of protein and energy seems to be limited by availability [12]; therefore, there are minimal benefits for dairy cows when using the same nitrogen source (pressed soybean meal and soybean meal) [16]. The above mixed results do not necessarily imply that balancing rumen-degradable starch and protein has no benefit for microbial protein synthesis in ruminants.

Starch is a common source of non-fiber carbohydrates for animals during the finishing period, often provided by grains. Corn and wheat are grains with the highest yields in the North China Plain [17]. The content of rumen-degradable starch (RDS) in wheat is higher than that in corn because they have different starch–protein matrices and starch granules, and their ruminal digestion rates are 78.9% and 54.1%, respectively [18]. In recent years, the total wheat produced has still been greater than the total wheat consumed by humans, and surplus wheat has been widely used as an energy source in livestock diets [19]. Due to its high rumen degradation rate, it is often used as a raw material for diets with a high RDS content [20,21]. As reported in many other studies, increased RDS (replacing corn with 35–40% DM wheat) in the diet can improve the growth performance of lambs [22], decrease the ruminal NH_3_-N concentration [23], enhance the richness of ruminal microbes [21], and increase the duodenal flow of microbial N [24]. The interactions of RDP and RDS in the diet should be considered when formulating diets for ruminants. However, to the best of our knowledge, to date, most studies on AH and AS have focused on rumen fermentation and nitrogen efficiency, and very few have been conducted using metabolomics to analyze the metabolic pathways, and it is unknown whether increased RDS in the diet can improve nitrogen efficiency when fed AH or AS. Therefore, the objective of this study was to determine how the forage type of alfalfa (AH vs. AS) and levels of RDS (low RDS vs. high RDS) affect (i) growth performance, rumen fermentation, and enzymatic activity; (ii) rumen epithelial morphology and urea transport proteins; and (iii) host rumen bacterial composition and metabolome. We hypothesized that the forage type of alfalfa and RDS would interact, with the RDS level increasing ruminal microbial protein synthesis and growth performance more when fed AS. In addition, we expected alfalfa type and RDS to affect rumen bacterial community and metabolome, with greater bacteria richness, increasing the amino acid biosynthesis of rumen bacteria as well as the expression of the urea transporter.

## 2. Materials and Methods

### 2.1. Animal and Experiment Design

Thirty-two Suffolk female sheep of similar weights (initial weight 27.28 ± 3.4 kg) aged three months were randomly assigned in a complete randomized block design to a 2 × 2 factorial arrangement of treatments (*n* = 8 per treatment). Alfalfa hay (AH) and alfalfa silage (AS) were determined based on the results of Farahani et al. [25]. The rumen-degradable starch (RDS) level was determined based on the results of other research [22] and in vitro experiments (Supplementary File S1, in vitro experiment). Thus, 4 treatments were evaluated: alfalfa hay and low RDS (AHLR); alfalfa hay and high RDS (AHHR); alfalfa silage and low RDS (ASLR); and alfalfa silage and high RDS (ASHR). Treatment diets were formulated to be isocaloric and isonitrogenous and met the NYT816-2021 recommendations (Table 1). Sheep were housed in an outdoor rearing system in individual pens (1.0 × 1.0 m^2^) bedded with sand. The experimental period was 75 d, including a 15 d transition period and a 60 d trial period. Sheep were fed twice daily at 09 and 16 h as TMR for ad libitum intake.

### 2.2. Silage Production and Cereal Grain Processing

The alfalfa was collected on the Hohhot, Inner Mongolia, China. The second cut of pure alfalfa plants (cultivar WL354) was harvested at the early bud stage of maturity and used for ensiling or barn drying. The drying of alfalfa mass is performed twice with a rake to smoothly move the curing hay, well-aerated windrow. From cutting to baling, drying has lasted a full 4 days, drying down to a moisture content of 18–20% before baling the hay. Fresh alfalfa (geometric mean cut length of 2.0 ± 0.1 cm) was equally spread on a white plastic sheet in the shade to low-intensity wilting treatment until DM concentrations of 400 g/kg were reached. It was then packed into the white plastic sheet, vacuum sealed, and stored with the RDP of alfalfa silage using a near-infrared reflectance spectroscopy estimate [26] (Supplementary File S2, Table S1).

Corn, wheat, soybean meal, wheat bran, soybean oil, calcium hydrogen phosphates, limestone, salt, and premix were obtained from a local feed mill. The above ingredients were prepared into concentrated particles according to Table 1.

### 2.3. Intake, Growth Performance, and Rumen Sample Collections

Daily dry matter intake (DMI) per sheep was calculated using amounts of feed offered and refused throughout the experiment. Sheep were weighed at the beginning, and at the end of the experiment, the sheep were weighed and then slaughtered using a captive bolt gun stunning method after the morning feeding for 3 h. Following slaughter, the rumen was taken out immediately and sampled. The rumen epithelial tissue was fixed using a 4% paraformaldehyde solution. Then, on the paraffin section, staining was performed, and the rumen papilla morphology was measured with a microscope. Another rumen epithelial tissue was separated from the muscle layer, rinsed with 0.9% Nacl solution, and put into a liquid nitrogen tank for storage for RNA extraction. Then, the rumen liquid and solid mixture were filtered into a sterile tube with four layers of gauze and frozen with liquid nitrogen. Samples were stored at −80 °C for the measurement of rumen digestive enzymes, fermentation parameters, metagenomics, and metabolomics [27]. After slaughter, the internal organs, head, and hooves were removed, the hot carcasses were weighed, and the dressing percentage was calculated.

The rumen fluid was then transferred into a micro-centrifuge tube and centrifuged (3000 r/min^−1^) to separate the upper clear fluid, which was used for measuring fermentation parameters. The VFA content was analyzed by gas chromatography (6890N, Agilent Technologies Inc., Santa Clara, CA, USA). The NH_3_-N and BCP concentrations were assessed by a spectrometer using colorimetry and a coomath bright blue process [28], respectively. The rumen content mixture was homogenized using an ultrasonic disintegrator to obtain a homogenization buffer. The supernatant was then collected by centrifuging, and the total amino acids (TAAs), lactic acid, α-amylase, cellulase activity, and urase activity were determined according to the instructions of the kit (jiancheng Biotechnology Co., Ltd., Nanjing, China).

### 2.4. Rumen Epithelial Fluorescent Quantitative PCR, Western Blotting, and Immunofluorescence Staining

Total RNA in the rumen epithelial was acquired using Trizol reagent (Accurate Biotechnology Co., Ltd., Changsha, China), and the total RNA samples were quantitatively and qualitatively evaluated with a spectrophotometer (Pultton P200 CM, San Jose, CA, USA) to ascertain the absorbance ratio at 260 and 280 nm. Then, the total RNA was processed with DNaseI (TaKaRa Biotechnology Co., Ltd., Dalian, China) to eliminate any genomic DNA contamination. Using the TB^®^ Green qPCR method along with a Prime Script RT™ Master Mix kit (TaKaRa Biotechnology Co., Ltd., Dalian, China) on LifeECO (Bori Technology Co., Ltd., Hangzhou, China), total RNA was converted to cDNA. The reactions were incubated at 37 °C for 15 min, followed by 5 s at 85 °C. Quantification of the cDNA transcript was performed using a qPCR TB Green Kit with the gene-specific primer on the LightCycler 96 real-time PCR system. qRT-PCR was performed using a 20 μL reaction mixture, and this mixture comprised 10 μL of TB green mix, 1.6 μL of each forward primer (with a concentration of 0.8 μM) and reverse primer (also at 0.8 μM), 2 μL of cDNA, and 6.4 μL of nuclease-free water with cycling conditions of 95 °C for 30 s and 60 °C for 30 s (30 cycles). The optimum annealing temperatures for different genes (Supplementary File S2, Table S2) were designed and synthesized by Shanghai Sangon Biotech (Shanghai, China). Glyceraldehyde-3-phosphate dehydrogenase (GAPDH) was used as a reference gene, and the relative transcript quantities in the mRNA expression level were calculated according to the 2^−ΔΔCT^ method.

Total protein in the rumen epithelial was acquired using a tissue protein extraction kit in accordance with the manufacturer’s instructions (Thermo Scientific, Shanghai, China). The total protein concentration was determined using a BCA protein assay (Beyotime, Biotech Inc., shanghai, China). Mix the protein sample and the protein loading buffer (ratio = 4:1) and heat the mixture (100 °C for 5 min) to denature the protein sample. Use the 10% SDS-PAGE gel rapid preparation kit for Western blotting. After electrophoresis (80 V for 40 min in the stacking gel and 120 V for 50 min in the resolving gel) and electroporation (110 V for 120 min), the converted membrane was blocked with 5% skimmed milk powder for 1 h and incubated with the primary antibody overnight at 4 °C (rabbit anti-AQP3, 1:500, bs-1253r, Bioss antibodies, Beijing, China). The converted membrane was incubated on a shaker in the dark with a secondary antibody for 1 h, and the converted membrane was processed with an ECL luminescent solution (G2161-200ML, Servicebio Technology Co., Wuhan, China). The protein separation membrane was scanned and analyzed using an image analyzer (SCG-W2000, Servicebio Technology Co., Wuhan, China).

For immunofluorescence, the permeabilization of rumen tissue slices was achieved using 0.3% Triton X-100 in PBS. The slices were blocked with 10% donkey serum (G1217, Servicebio Technology Co., Wuhan, China) at room temperature for 40 min and added to the primary antibody, and they were incubated overnight at 4 °C. The antibodies used included rabbit anti-UT-B (1:1000; 25962-1-AP, Proteintech Group, Inc., Wuhan, China), rabbit anti-AQP3 (1:500; bs-1253r, Bioss antibodies, Beijing, China), rabbit anti-AQP7 (1:500; 25131-1-AP; Proteintech Group, Inc., Wuhan, China), and rabbit anti-AQP10 (1:500; Q96PS8; Servicebio Technology Co., Wuhan, China). Subsequently, the sections were washed with PBS and incubated with secondary antibodies (1:400, GB25303, Servicebio Technology Co., Wuhan, China) at room temperature in the dark for 1 h, followed by DAPI (G1012, Servicebio Technology Co., Wuhan, China) staining. The images were analyzed with a microscope (Nikon Eclipse C1, Tokyo, Japan) and a scanner (3DHISTECH, Pannoramic, Tokyo, Japan).

### 2.5. Rumen Bacterial DNA Extraction and Analysis

Total microbial genomic DNA was extracted from rumen contents using the E.Z.N.A.^®^ soil DNA Kit (Omega Bio-tek, Norcross, GA, USA), following the manufacturer’s instructions. The hypervariable region V3-V4 of the bacterial 16S rRNA gene was amplified with primer pairs 338F (5′-ACTCCTACGGGAGGCAGCAG-3′) and 806R (5′-GGACTACHVGGGTWTCTAAT-3′) by T100 Thermal Cycler PCR thermos cycler (BIO-RAD, Hercules, CA, USA) [29]. As part of the PCR analysis process, the PCR products were mixed and recovered on 2% agarose gel, purified by the AxyPrep DNA Gel Extraction Kit (Axygen Biosciences, Union City, CA, USA), and homogenized and quantified by 2% agarose gel electrophoresis and Quantus™ Fluorometer (Promega Biotech Co., Ltd., Fitchburg, WI, USA). The sequencing library was prepared using the NEXTFLEX Rapid DNA-Seq Kit, and the qualified library was sequenced with the Illumina Miseq PE300 platform (Illumina, San Diego, GA, USA).

The raw sequenced sequences were quality controlled by fastp version 0.19.6 and merged by FLASH version 1.2.7. Operational taxonomic unit (OTU) clustering was conducted at a 97% sequence similarity threshold using the Uparse software (https://drive5.com/uparse/, accessed on 17 October 2023). All optimized sequences were mapped to the OTU representative sequences, and those with 97% or more similarity to the OTU representative sequences were selected to generate the OTU table [30].

Bioinformatic analysis of the rumen bacterial was carried out using the Majorbio Cloud platform (https://cloud.majorbio.com, accessed on 17 October 2023). Based on the OTU information, rarefaction curves, and alpha diversity indices, including observed OTUs, the Evenness, Chao1 richness, and Shannon index were calculated with Mothur v1.30.1. Evenness describes the relative abundance of the different species making up the richness [31].

### 2.6. Analysis of Untargeted Metabolomics Data Preprocessing and Multivariate Statistical Analysis

The sample treatment, detection procedures, and raw data processing of rumen fluid metabolome are as shown by Zhang et al. [30]. Briefly, a 100 μL rumen liquid sample was mixed with 400 μL of an acetonitrile:methanol (1:1) solution containing 0.02 mg/mL internal standard (L-2-chlorophenylalanine) to extract metabolites. The mixture was mixed using a vortex for 30 s and then sonicated at low temperature for 30 min at 5 °C with a frequency of 40 KHz. Subsequently, the proteins were precipitated for 30 min at −20 °C. After centrifuging at 15 min (4 °C, 13,000× *g*), the supernatant was transferred to sample vials for LC-MS/MS analysis.

The LC-MS/MS analysis of the sample was conducted on a Thermo UHPLC-Q Exactive system equipped with an ACQUITY HSS T3 column (100 mm × 2.1 mm i.d., 1.8 μm; Waters, MA, USA) at Majorbio Bio-Pharm Technology Co., Ltd. (Shanghai, China). The mobile phases consisted of 0.1% formic acid in water:acetonitrile (95:5, *v*/*v*) (solvent A) and 0.1% formic acid in acetonitrile:isopropanol:water (47.5:47.5, *v*/*v*) (solvent B). The flow rate was 0.40 mL/min, and the column temperature was 40 °C. (MS conditions: The UPLC system was coupled to a Thermo UHPLC-Q Exactive Mass Spectrometer equipped with an electrospray ionization (ESI) source operating in positive mode and negative mode. The optimal conditions were set as follows: source temperature at 400 °C; sheath gas flow rate at 40 arb; Aux gas flow rate at 10 arb; ion-spray voltage floating (ISVF) at −2800 V in negative mode and 3500 V in positive mode, respectively; normalized collision energy, 20–40–60 V rolling for MS/MS. Full MS resolution was 70,000, and MS/MS resolution was 17,500. Data acquisition was performed with the Data-Dependent Acquisition (DDA) mode. The detection was carried out over a mass range of 70–1050 *m*/*z*).

The pretreatment of LC/MS raw data was performed by Progenesis QI (v3.0) (Waters Corporation, Milford, CT, USA) software, and a three-dimensional data matrix in CSV format was exported. The information in this three-dimensional matrix included sample information, metabolite name, and mass spectral response intensity. Internal standard peaks, as well as any known false positive peaks (including noise, column bleed, and derivatized reagent peaks), were removed from the data matrix, and they were redundant and peak pooled. At the same time, the metabolites were identified by searching databases, and the main databases were the HMDB (http://www.hmdb.ca/, accessed on 19 September 2023), Metlin (https://metlin.scripps.edu/, accessed on 19 September 2023), and Majorbio Database. The data were analyzed through the free online platform of the majorbio cloud platform (cloud.majorbio.com, accessed on 19 September 2023). Metabolic features detected at least 80% in any set of samples were retained. After filtering, minimum metabolite values were imputed for specific samples in which the metabolite levels fell below the lower limit of quantitation, and each metabolic feature was normalized by sum. To reduce the errors caused by sample preparation and instrument instability, the response intensity of the sample mass spectrum peaks was normalized by the sum normalization method, and then the normalized data matrix was obtained. Meanwhile, variables with relative standard deviation (RSD) > 30% of QC samples were removed, and log10 processing was performed to obtain the final data matrix for subsequent analysis.

After data preprocessing, variance analysis was conducted on the matrix file to identify differential metabolites (DMs). These DMs were determined through pairwise comparisons between AHLR and AHHR, AHLR and ASLR, and AHLR and ASHR. Utilizing the R package “ropls” (version 1.6.2), we performed principal component analysis (PCA) and orthogonal partial least squares discriminant analysis (OPLS-DA), followed by a 7-cycle interactive validation to assess model stability. Metabolites with VIP > 1 and *p* < 0.05, based on the variable importance in projection (VIP) derived from the OPLS-DA model and *p*-values from Student’s *t*-test, were considered significantly different. The DMs between the two groups were mapped to their biochemical pathways through metabolic enrichment and pathway analysis using the KEGG database (http://www.genome.jp/kegg/, accessed on 27 December 2023). These metabolites were classified according to the pathways they participated in or the functions they served. The metabolome map displays all matched pathways, considering *p*-values from pathway enrichment analysis and pathway impact values from pathway topology analysis. Pathways with both high impact values and significant *p*-values were identified as key pathways.

### 2.7. Data Processing and Analysis

Phenotypic data (growth performance, rumen fermentation parameters, and papillae morphology) were analyzed using a one-way ANOVA procedure with post hoc Tukey tests, along with the mixed model procedure of SAS (Proc Mixed; SAS, 1996). The model included forage (AS:AH), RDS (LR:HR), and the two-way interaction between forage and RDS. Spearman’s correlation analysis was conducted using the CORR procedure of SAS. Significance was declared at *p* < 0.05, and a tendency was declared at 0.05 < *p* < 0.10.

Correlation analysis between rumen bacterial taxa, alpha diversity indices, rumen fermentation parameters, and phenotypic data was performed using Spearman’s rank correlation, with a coefficient of >|0.4|, *p* <  0.05 considered significant. The different rumen metabolites with a VIP of >1.5 and *p* < 0.05 and different microbial genera (top ten microbial genera in relative abundance) were performed using Spearman’s rank correlation, with a coefficient of >|0.4|, as well as *p* <  0.05, which was considered significant.

## 3. Results

### 3.1. Growth Performance, Stomach Weight, Rumen Fermentation Parameters, and Rumen Papillae Morphology

For the HR diet, DMI and average daily gain (ADG) increased (*p* < 0.05), resulting in a lower F:G (*p* < 0.05) and a tendency for the dressing percentage to be lower (*p* < 0.10) compared to sheep fed the LR diet. The F:G tended to be lower (*p* < 0.10) in the AS diet. In addition, rumen weight was greater in the HR diet (*p* < 0.05), and abomasal weight was affected by a forage × RDS interaction (*p* < 0.10) (Table 2).

The NH_3_-N concentration in rumen fluid was greater in sheep fed AS than the AH diet (*p* < 0.01) and greater for the HR diet relative to the LR diet (*p* < 0.01) and was affected by a forage × RDS interaction (*p* < 0.01). In contrast, the concentration of BCP showed opposite trends (forage *p* < 0.01, grain *p* < 0.01, forage × grain *p* < 0.10). The TAA concentration was greater (*p* < 0.05), and α-amylase activity tended to be greater (*p* < 0.10) in the HR diet. Urase activity was greater in sheep fed AS compared with those fed AH (*p* < 0.05). The molar proportions of isobutyrate (*p* < 0.01) and valerate (*p* < 0.05) were greater in the AS diet than AH diet, while lactic acid concentration was decreased (*p* < 0.05) for the AS diet compared to the AH diet and decreased for the HR diet relative to the LR diet (*p* < 0.01) (Table 3).

**Figure 1:**
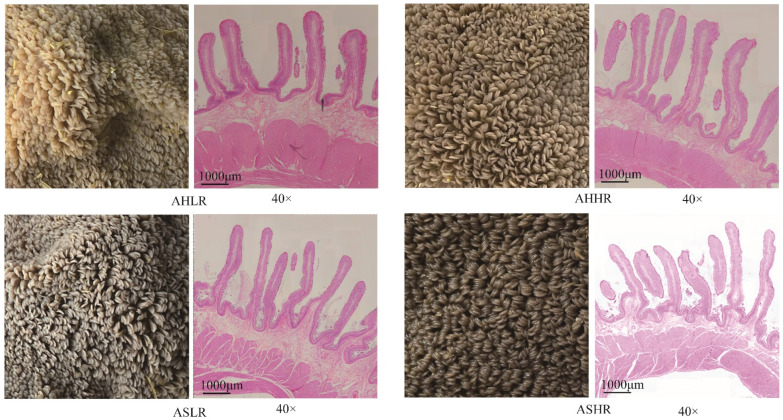
Rumen epithelium photograph and HE staining of rumen epithelia (40×). AHLR: alfalfa hay and low RDS, AHHR: alfalfa hay and high RDS, ASLR: alfalfa silage and low RDS, ASHR: alfalfa silage and high RDS.

As shown in the rumen epithelium (Table 3, Figure 1), sheep fed the AH diet results in a straw-yellow coloration of the rumen epithelium, whereas AS feeding leads to a darker, almost black coloration. HE indicates that rumen papillae tended to be taller (*p* < 0.10), and papillae width was lower (*p* < 0.05) in sheep fed the AS diet than the AH diet, it but had no effect on their surface. Lamina propria thickness and muscle layer thickness were not affected by forage, RDS, or the interaction between forage and RDS.

**Figure 2:**
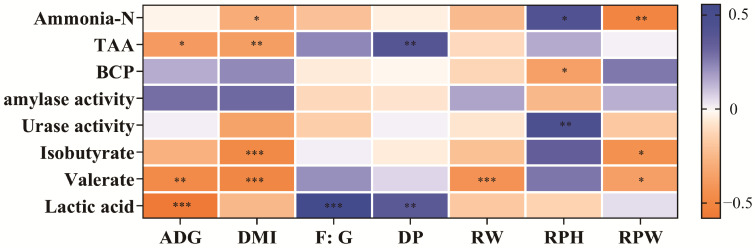
Rumen fermentation and its relationship with growth performance. TAA: total amino acid. BCP: bacterial protein. DP: dressing percentage. RW: rumen weight. RPH: rumen papillae height. RPW: rumen papillae width. “*”, “**”, and “***” mean significant differences and “*p* < 0.05”, “*p* < 0.01”, and “*p* < 0.001”, respectively.

Spearman’s correlation analysis was performed between growth performance and rumen fermentation parameters (Figure 2). The correlation results showed that rumen total amino acids, isobutyrate, valerate, and lactic acid concentrations were negatively correlated with ADG, DMI, and rumen weight, and total amino acids and lactic acid were positively correlated with the F:G at a dressing percentage (r  >  0.2, *p*  <  0.05). NH_3_-N concentrations and urase activity were positively correlated with rumen papillae height, while isobutyrate and valerate concentrations were negatively correlated with rumen papillae width (r  >  0.2, *p* < 0.05).

### 3.2. AQP and UT-B mRNA and Protein Abundance in Ruminal Epithelium

**Figure 3:**
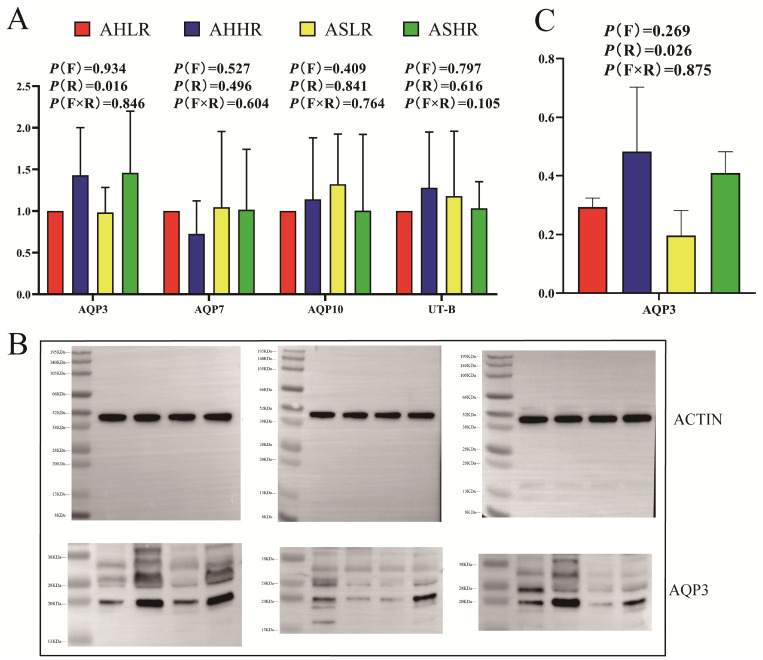
The mRNA and protein expression of urea transporters in rumen epithelium. (**A**) The mRNA expression of urea transporters in rumen epithelium. (**B**) WB of AQP3 in rumen epithelium. (**C**) The protein expression of AQP3 in rumen epithelium. AHLR: alfalfa hay and low RDS, AHHR: alfalfa hay and high RDS, ASLR: alfalfa silage and low RDS, ASHR: alfalfa silage and high RDS. P (F) = alfalfa hay versus alfalfa silage (AH vs. AS); P (R) = low RDS versus high RDS (LR vs. HR); P (F × R) = forage by RDS interaction.

**Figure 4:**
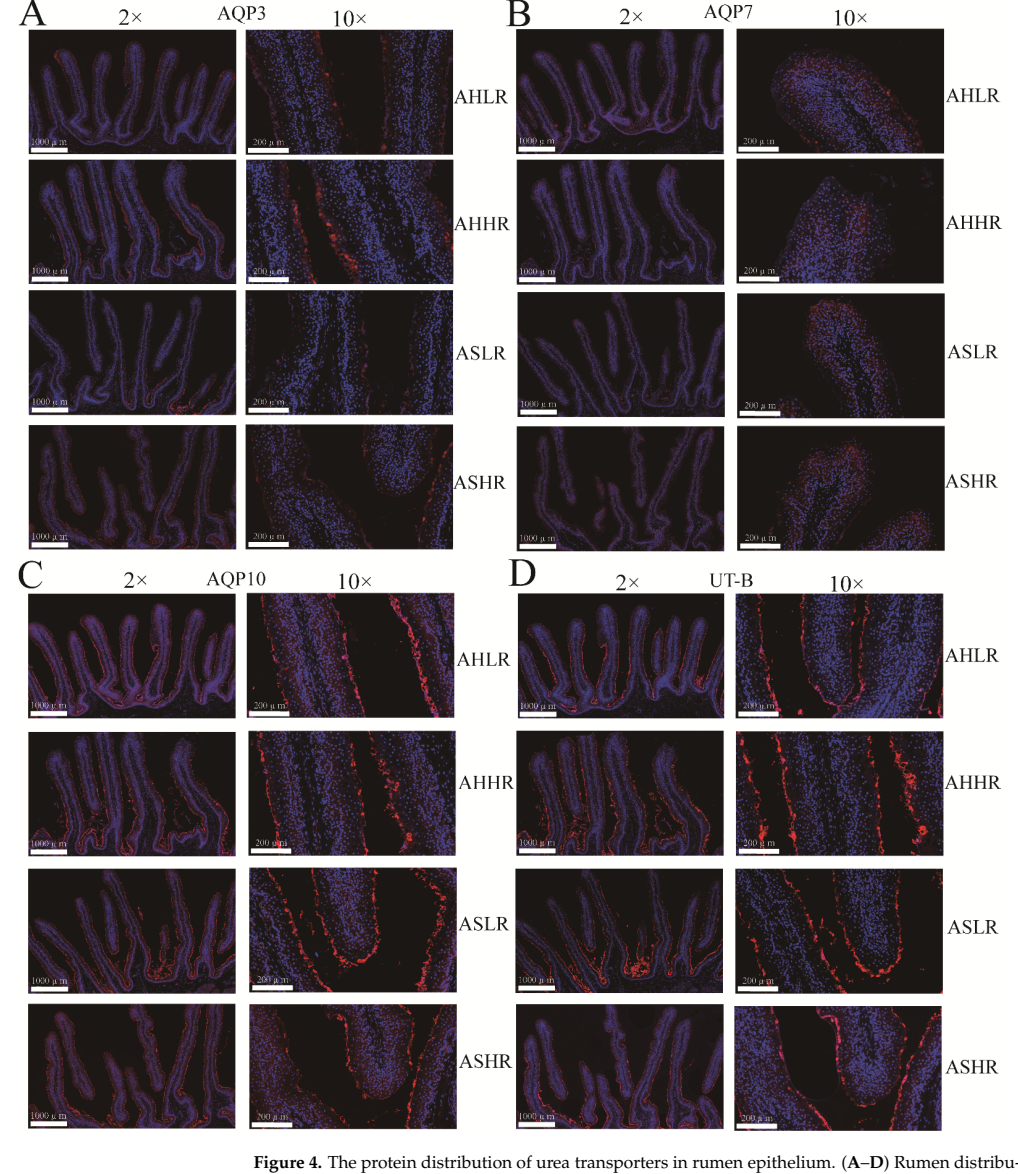
The protein distribution of urea transporters in rumen epithelium. (**A**–**D**) Rumen distribution of AQP3, AQP7, AQP10, and UT-B protein, respectively. AHLR: alfalfa hay and low RDS, AHHR: alfalfa hay and high RDS, ASLR: alfalfa silage and low RDS, ASHR: alfalfa silage and high RDS.

The results of real-time PCR and Western blot showed that the upregulation (*p* < 0.05) of AQP3 was observed in the rumen epithelium of sheep fed the HR diet compared to those fed the LR diet (Figure 3A–C). Furthermore, the immunofluorescence results showed that both AQP and UT-B were expressed on the rumen epithelium, AQP3 and UT-B were mainly distributed in the stratum corneum, and AQP7 and AQP10 were distributed throughout all the layers of rumen epithelium (stratum corneum, stratum granulosum, stratum spinosum, and stratum basale). AQP3 protein in the rumen epithelium in the HR diet was upregulated compared with that in the LR diet (Figure 4A–D).

### 3.3. Ruminal Bacterial Communities

**Figure 5:**
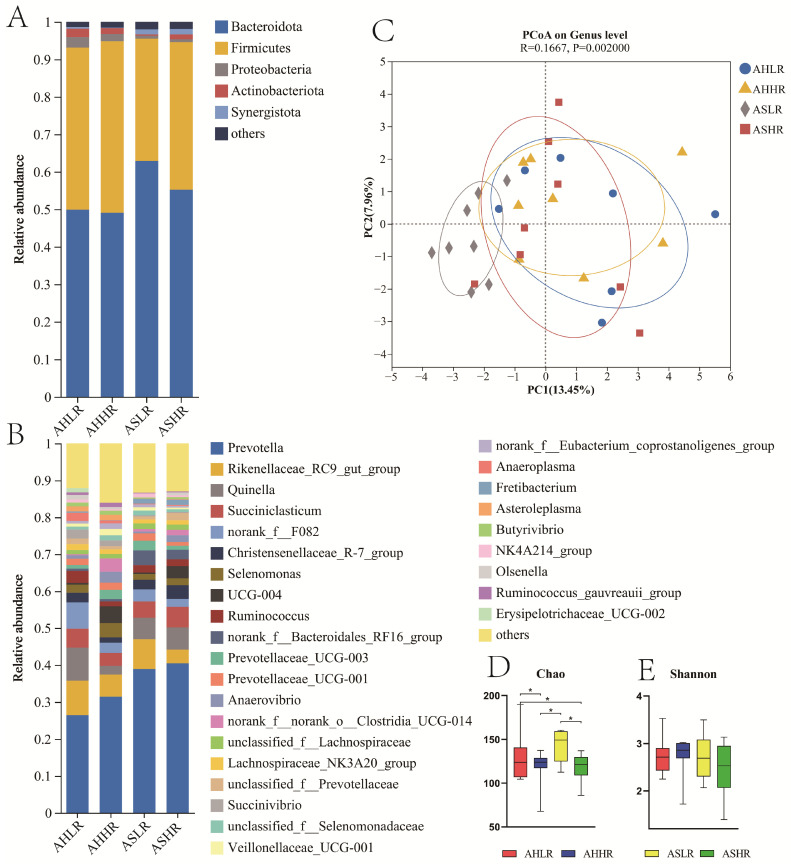
Effect of rumen-degradable starch on alpha diversity indexes of rumen bacteria in sheep fed AH or AS. (**A**) Bacterial taxa averaged under phyla. (**B**) Bacterial taxa averaged under genera. (**C**) Principal coordinate analysis (PCoA) of beta diversity. (**D**) Chao index of alpha diversity. (**E**) Shannon index of alpha diversity. AHLR: alfalfa hay and low RDS, AHHR: alfalfa hay and high RDS, ASLR: alfalfa silage and low RDS, ASHR: alfalfa silage and high RDS. “*” means a significant difference “*p* < 0.05”.

The analysis of bacterial alpha diversity showed that the Chao index for sheep fed AH was lower (*p* = 0.021), while the bacteria alpha diversity was not impacted (*p* ≥ 0.1) by forage and RDS interaction (Figure 5D,E). Principal coordinate analysis (PCoA) was performed based on the binary Euclidean dissimilarities metric in the bacterial community. Our bacterial beta diversity analysis showed an obvious separation between the ASLR with the other three groups (Figure 5C).

Five bacteria phyla were identified with relative abundances of more than 0.1% in at least one group. The dominant bacterial phyla in the rumen were *Bacteroidota* (AHLR: 49.89%, AHHR 49.07%, ASLR: 62.91%, ASHR: 55.23%) and *Firmicutes* (43.26%, 45.76%, 32.57%, 39.37%) (Figure 5A). Sixteen bacteria genera were identified with relative abundances of more than 2.0% in at least one group. The dominant bacteria in the AHLR, AHHR, ASLR, and ASHR groups were *Prevotella* (AHLR: 26.43%, AHHR: 31.43%, ASLR: 38.88%, ASHR: 40.45%), *Rikenellaceae_RC9_gut_group* (AHLR: 9.31%, AHHR: 5.98%, ASLR: 8.10%, ASHR: 5.54%), *Quinella* (AHLR: 8.93%, AHHR: 2.33%, ASLR: 5.82%, ASHR: 6.04%), *Succiniclasticum* (AHLR: 7.12%, AHHR: 3.53%, ASLR: 4.41%, ASHR: 5.54%), and *norank_f__F082* (AHLR: 5.15%, AHHR: 2.75%, ASLR: 3.28%, ASHR: 2.18%) (Figure 5B). Although these genera were dominant, their abundances did not differ significantly among the four groups.

### 3.4. Ruminal Metabolomics Analysis

**Figure 6:**
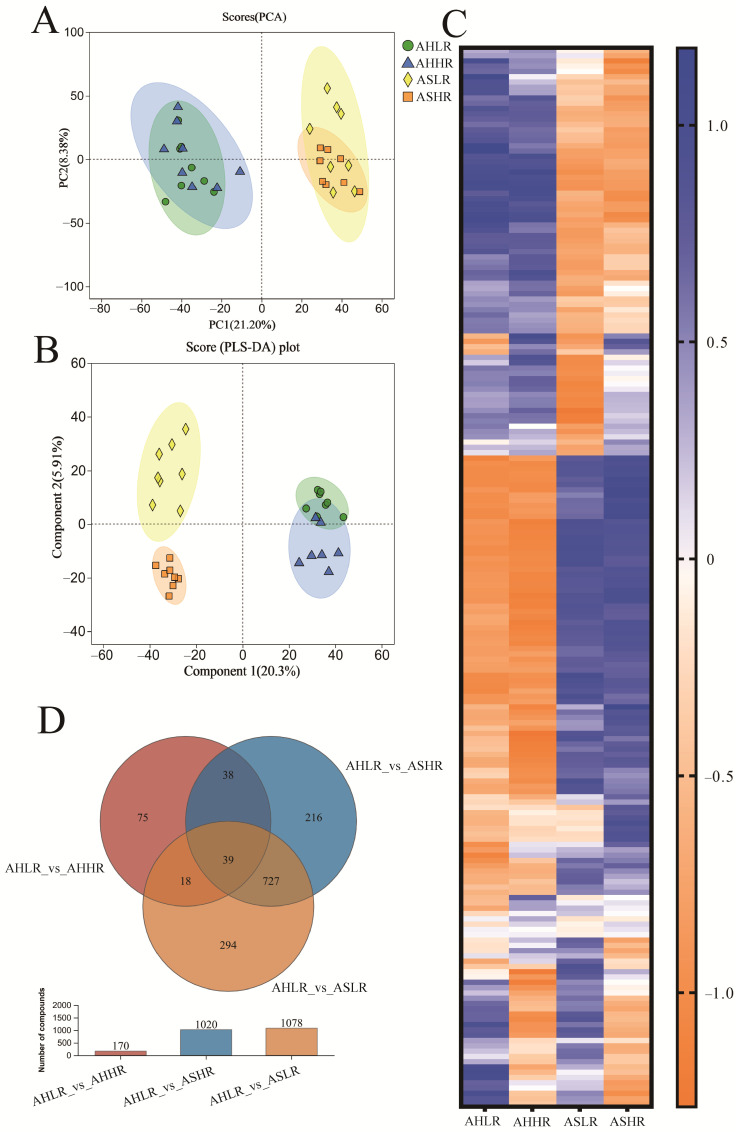
Rumen metabolome analysis. (**A**) Principal coordinate analysis (PCA). (**B**) Partial least square discriminant analysis (PLS-DA) score. (**C**) Heatmap of the top 200 differential metabolites in rumen fluids among AHLR, AHHR, ASLR, and ASHR. (**D**) Venn diagrams show the number of common and unique features of the three comparison pairs. AHLR: alfalfa hay and low RDS, AHHR: alfalfa hay and high RDS, ASLR: alfalfa silage and low RDS, ASHR: alfalfa silage and high RDS.

A total of 3330 compounds were identified in the rumen metabolites. The PCA score map showed that feeding AH or AS could separate rumen metabolites, but feeding LRDS or HRDS resulted in mostly overlapping distributions in the score plots for all sheep (Figure 6A). However, the PLS-DA score plot (Figure 6B) showed clear distinguishability among the rumen metabolites of AHLR, AHHR, ASLR, and ASHR. This clear separation was also demonstrated in the heatmap among the four groups (top 200 differential metabolites, Figure 6C). Of the 3330 metabolites, 1407 important metabolites (*p* < 0.05 and VIP > 1) were identified, with 170, 1078, and 1020 differential metabolites in AHLR vs. AHHR, AHLR vs. ASLR, and AHLR vs. ASHR, respectively. Thirty-nine metabolites were common across the three comparison pairs, while the majority of the differential metabolites (727) were common between AHLR vs. ASLR and AHLR vs. ASHR. These 1407 differential metabolites have been used for pathway analysis based on KEGG modules (Figure 6D) (Supplementary File S3: Tables S3–S5).

**Figure 7:**
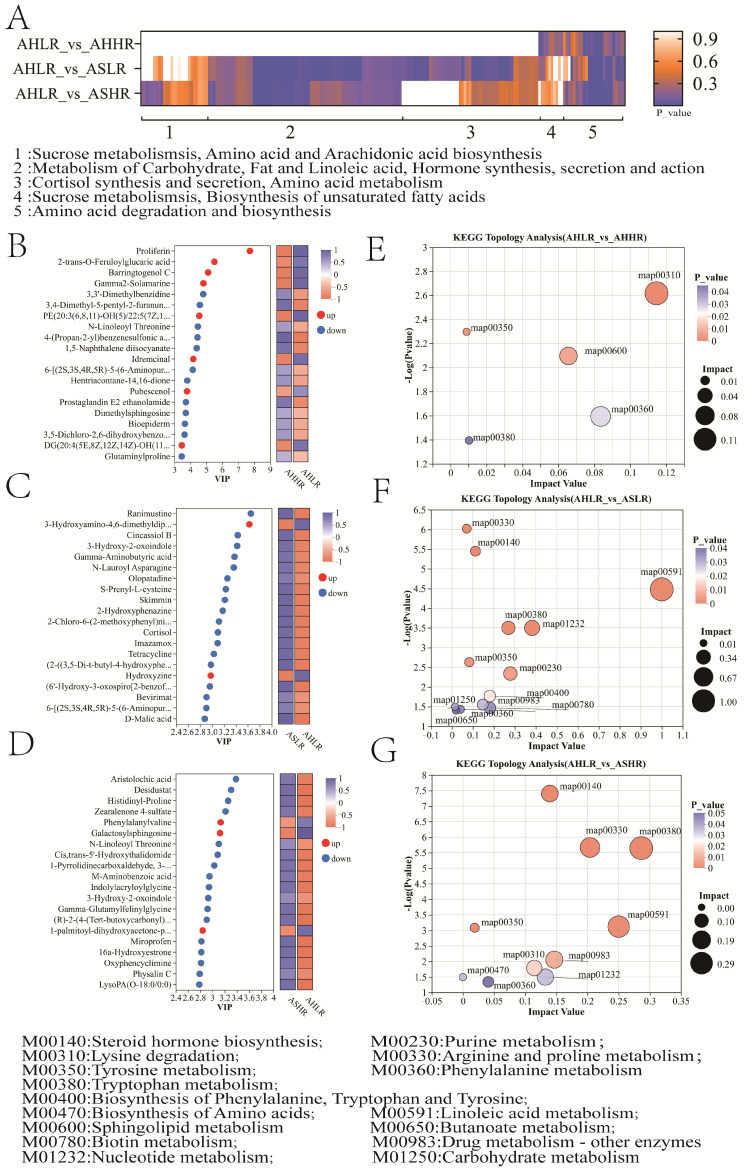
Differential metabolic pathway analysis. (**A**) The heatmap of differential metabolites among three comparison pairs and the biochemical pathways involved. (**B**) The variable importance in projection (VIP) scores in the three comparison pairs (AHLR vs. AHHR, AHLR vs. ASLR, and AHLR vs. ASHR), respectively (**B**–**D**). Top 15 significant features (*p* < 0.05) based on VIP scores. Relevant metabolic pathways involved in the divergence among three comparison pairs (AHLR vs. AHHR, AHLR vs. ASLR, and AHLR vs. ASHR), respectively (**E**–**G**). AHLR: alfalfa hay and low RDS, AHHR: alfalfa hay and high RDS, ASLR: alfalfa silage and low RDS, ASHR: alfalfa silage and high RDS.

The biochemical pathways involved in differential metabolites differed among the three comparison pairs (Figure 7A). In the AHLR vs. ASLR comparison, metabolic pathways involved fat and amino acid metabolism, and in the AHLR vs. ASHR comparison, metabolic pathways involved unsaturated fatty acids and amino acid biosynthesis. The mutual pathway among AHLR vs. AHHR, AHLR vs. ASLR, and AHLR vs. ASHR was sucrose metabolisms, unsaturated fatty acid biosynthesis, and amino acid degradation and biosynthesis. Subsequently, we identified 20 features in each of the comparison pairs (AHLR vs. AHHR, AHLR vs. ASLR, and AHLR vs. ASHR), respectively, based on variable importance in projection (VIP) scores (>2.0), SAM, and/or ANOVA (Figure 7B–D). The significantly different metabolites (VIP > 1, *p* < 0.05) among the comparison pairs were mapped into their biochemical pathways through metabolic enrichment and pathway analysis based on the KEGG database, *p* < 0.05, and were regarded as key pathways. In the AHHR group compared to the AHLR group, two metabolic pathways, lysine degradation and sphingolipid metabolism, were upregulated, while three metabolic pathways, phenylalanine metabolism, tyrosine metabolism, and tryptophan metabolism, were downregulated (Figure 7E). In the ASLR group compared to the AHLR group, two metabolic pathways, tryptophan metabolism and steroid hormone biosynthesis, were upregulated, while twelve metabolic pathways, linoleic acid metabolism, nucleotide metabolism, purine metabolism, biotin metabolism, phenylalanine tyrosine and tryptophan biosynthesis, drug metabolism–other enzymes, tyrosine metabolism, arginine and proline metabolism, phenylalanine metabolism, butanoate metabolism, and biosynthesis of nucleotide sugars, were downregulated (Figure 7F). In the ASHR group compared to the AHLR group, two metabolic pathways, steroid hormone biosynthesis and tryptophan metabolism, were upregulated, nine metabolic pathways, linoleic acid metabolism, arginine and proline metabolism, drug metabolism–other enzymes, steroid hormone biosynthesis, nucleotide metabolism, lysine degradation, phenylalanine metabolism, tyrosine metabolism, and D-amino acid metabolism, were downregulated (Figure 7G).

**Figure 8:**
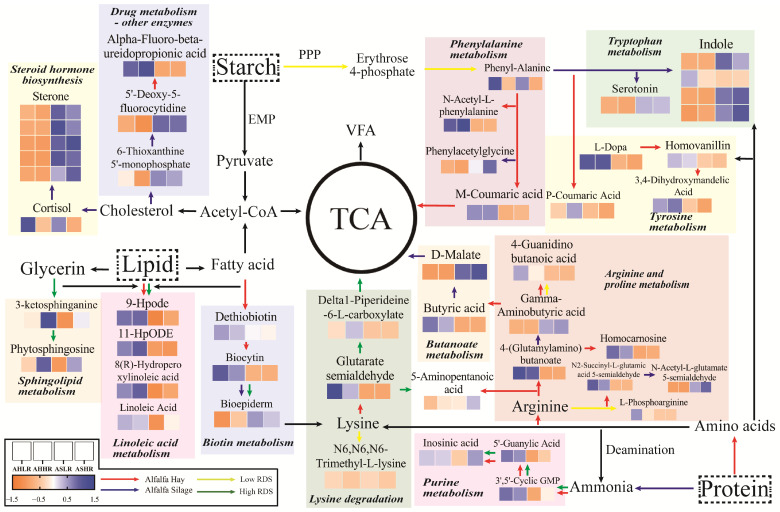
The metabolic pathway reconstruction graphs of the starch, nitrogen, and lipid in the rumen of sheep fed different forage and grain sources. AHLR: alfalfa hay and low RDS, AHHR: alfalfa hay and high RDS, ASLR: alfalfa silage and low RDS, ASHR: alfalfa silage and high RDS. The red line, blue line, yellow line, green line, and black line represent the metabolic pathway of AH rich, AS rich, low RDS rich, high RDS rich, and not enriched, respectively. Each heatmap indicates different metabolites associated with metabolic pathways, and each is presented in Supplementary File S3, Table S6 in detail. PPP: pentose phosphate pathway. EMP: Embden–Meyerhof pathway. VFA: volatile fatty acid. TCA: tricarboxylic acid cycle. MCP: bacterial protein.

We reconstructed metabolic pathways based on the significantly altered KOs in the rumen (Figure 8). The results showed that the HRDS diet reduced rumen microbial tyrosine, phenylalanine, and tryptophan biosynthesis pathways and enriched lysine degradation and sphingolipid metabolism pathways compared to the LRDS diet, indicating that high RDS stimulates microbial protein synthesis. Additionally, the rumen of sheep fed AS showed significant enrichment in tryptophan degradation and hormone synthesis pathways, while tyrosine, phenylalanine, and tryptophan degradation, butanoate metabolism, arginine, proline metabolism, and linoleic acid metabolism were reduced, indicating an increased degradation of AS protein to NPN and lipid degradation to cholesterol. After increasing RDS in the AS diet, the purine metabolism, linoleic acid metabolism, and amino acid synthesis pathways were slightly improved. These results suggested that the HRDS diet can stimulate microbial protein synthesis, thereby slightly improving amino acid metabolism and unsaturated fatty acid metabolism.

### 3.5. Phenotypic Traits, Microbial Genera, and Rumen Differential Metabolites and Their Relationships

**Figure 9:**
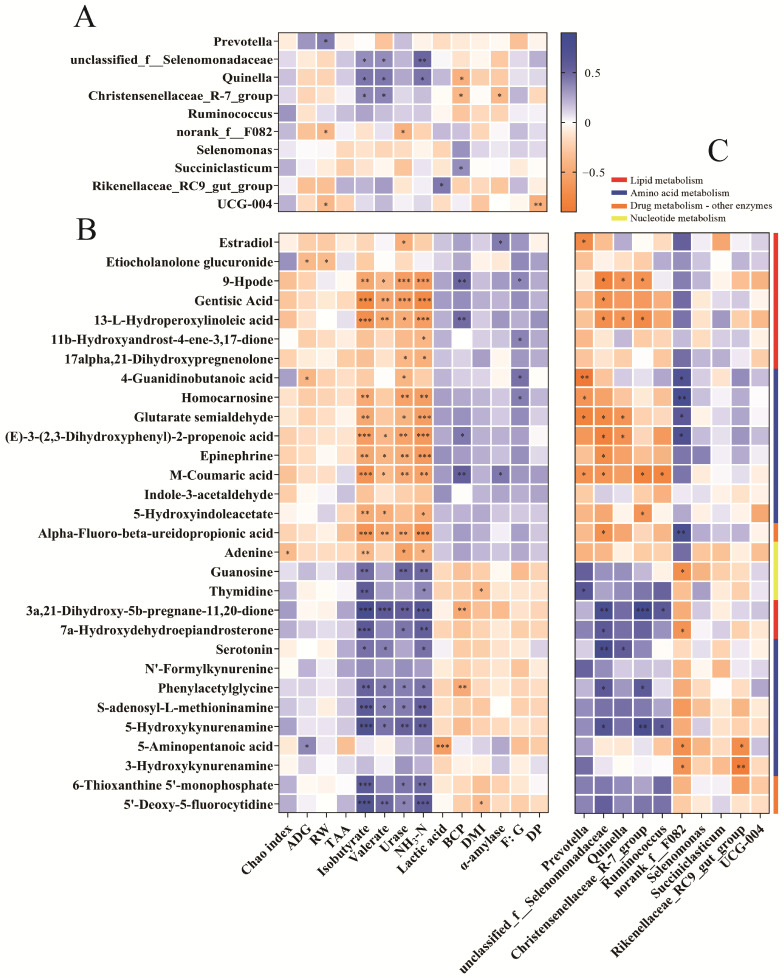
The relationship among significantly different growth performance and rumen fermentation parameters, microbial genera (top ten microbial genera in relative abundance), and rumen differential metabolites. (**A**) Spearman’s correlation analysis between rumen bacteria and phenotypic traits. (**B**) Spearman’s correlation analysis between rumen differential metabolites and phenotypic traits. (**C**) Spearman’s correlation analysis between rumen differential metabolites and bacteria. TAA: total amino acid; BCP: bacterial protein. DP: dressing percentage. RW: rumen weight. “*”, “**”, and “***” mean significant differences and “*p* < 0.05”, “*p* < 0.01”, and “*p* < 0.001”, respectively.

Spearman’s correlation analysis was performed among the significantly different phenotypic traits, microbial genera (top ten microbial genera in relative abundance), and rumen differential metabolites (top 30 differential metabolite correlations to phenotypic data, Figure 9). The correlation results revealed that the Chao index was positively correlated (*p* < 0.05) with adenine. ADG was negatively correlated (*p* < 0.05) with etiocholanolone glucuronide and 4-guanidinobutanoic acid and positively correlated (*p* < 0.05) with 5-aminopentanoic acid. RW was negatively correlated (*p* < 0.05) with *norank_f__F082, Rikenellaceae_RC9_gut_group*, and etiocholanolone glucuronide and positively correlated (*p* < 0.05) with *Prevotella*. Isobutyrate, valerate, urase, and, NH_3_-N were negatively correlated (*p* < 0.05) with 9-Hpode, gentisic acid, 13-L-Hydroperoxylinoleic acid, homocarnosine, glutarate semialdehyde, (E)-3-(2,3-Dihydroxyphenyl)-2-propenoic acid, epinephrine, M-Coumaric acid, 5-Hydroxyindoleacetate, Alpha–Fluoro–beta–ureidopropionic acid, and adenine and positively correlated (*p* < 0.05) with *unclassified_f__Selenomonadaceae, Quinella, Christensenellaceae_R-7_group*, guanosine, thymidine, 3a,21-Dihydroxy-5b-pregnane-11,20-dione, 7a-Hydroxydehydroepiandrosterone, serotonin, phenylacetylglycine, S-adenosyl-L-methioninamine, 5-Hydroxykynurenamine, 6-Thioxanthine 5′-monophosphate, and 5′-Deoxy-5-fluorocytidine. Lactic acid was negatively correlated (*p* < 0.05) with 3a,21-Dihydroxy-5b-pregnane-11,20-dione and phenylacetylglycine, and positively correlated (*p* < 0.05) with *Rikenellaceae_RC9_gut_group*. BCP was negatively correlated (*p* < 0.05) with *Quinella, Christensenellaceae_R-7_group*, and 5-aminopentanoic acid and positively correlated (*p* < 0.05) with *Succiniclasticum*, 9-Hpode, 13-L-Hydroperoxylinoleic acid, (E)-3-(2,3-Dihydroxyphenyl)-2-propenoic acid, and M-Coumaric acid. DMI was negatively correlated (*p* < 0.05) with thymidine and 5′-Deoxy-5-fluorocytidine. α-amylase was negatively correlated (*p* < 0.05) with *Christensenellaceae_R-7_group* and positively correlated (*p* < 0.05) with estradiol and M-Coumaric acid. The F:G was positively correlated (*p* < 0.05) with 9-Hpode, 11b-Hydroxyandrost-4-ene-3,17-dione, 4-Guanidinobutanoic acid, homocarnosine, and M-Coumaric acid. DP was negatively correlated (*p* < 0.05) with *UCG-004*.

## 4. Discussion

Feeding AS had no impact on the growth performance of sheep, as has been reported by others [7,32,33,34], but it led to a tendency for a reduction in the F:G. This might be due to the bio-fermentation process altering the physical characteristics of AS, whereby degrading hemicellulose produces more energy and results in a slightly higher digestibility. It has been suggested that the energy value (DM) from AS was approximately 1.24 times that of AH [35]. Additionally, growth performance was greater in sheep fed the HR diet compared with those fed the LR diet. In the present study, wheat was used as a 33% DM replacement for corn to increase the RDS content of the diet. Similarly, Karim et al. [22] reported that the gain of lambs improved when 35.3% DM wheat replaced maize. The reason for such variation could be that wheat contains more lysine [36,37]. Wheat starch has a higher proportion of amylopectin compared to corn, resulting in it being easier for it to bind with amylase for hydrolysis. Wheat starch was slightly more digestible in the total tract [38]. It has been suggested that reducing the ratio of amylose to amylopectin in the diet can improve the ADG of Qinchuan cattle [39]. Additionally, although the HR diet increased ADG, it decreased the dressing percentage and increased rumen weight in the present study. This is due to the increased amino acid consumption by microorganisms in the HR diet. It has been suggested that a high amylose/amylopectin ratio diet could reduce amino acid (AA) consumption in the intestine, allowing more AAs to enter the blood and maintain higher muscle protein synthesis [40,41]. These effects may further alter the amounts of fatty acids in muscle and fat [42], but they were not assessed in this paper. Furthermore, the HR diet had higher rumen weight, and it altered the site and extent of starch digestion and produced more ineffective weight gain.

The pH of healthy rumen ranges from 6.0 to 7.0, while a pH of 5.6–5.8 indicates a trend towards ruminal acidosis. It has been suggested that the inclusion level of wheat in the diet of ruminants should be limited to <40% DM, as it may reduce rumen pH and impair digestive function and production performance [43]. In the present study, rumen fluid pH was not affected by RDS level and was 5.9 in the LR diet, while it ranged from 5.7 to 5.8 in the HR diet. Therefore, the animals in this study were still in a healthy state at this time. Similarly, others [22,44] also reported that when 30% and 40% diet DM wheat replaced corn, the pH was between 5.8 and 6.0 respectively.

As expected, although all experimental diets were isonitrogenously formulated, feeding the AS diet resulted in excessive production of NH_3_-N in the rumen and reduced BCP synthesis in the present study. Our results were consistent with those of Vagnoni et al. [7], where concentrations of ruminal NH_3_ and total free AAs were higher in cows fed AS diets than cows fed AH diets. This reflected the extensive ruminal degradation of alfalfa silage CP, as well as the large amount of N present in the form of peptides and free AAs. Moate et al. [23] reported that replacing corn with wheat (43% of diet DM) results in a decrease in ruminal NH_3_-N concentration in dairy cows. Moreover, Plascencia et al. [24] also reported an increase in the omasum flow of microbial N when corn was substituted by wheat, indicating enhanced ruminal utilization of N due to rapid rumen starch fermentation. NH_3_-N accumulation was caused by the different degradability and degradation rates of AH and AS proteins in rumen [45]. Feeding the HR diet improved rumen BCP concentration and α-amylase activity and reduced NH_3_-N, total AAs, and lactic acid concentration. This indicates an increase in rapid starch degradation that also upregulates the microbial capture of peptide- and amino-N, thereby reducing their breakdown into NH_3_-N. Similarly, Shen et al. [20] reported an increase in amyloglucosidase in rumen with increasing RDS levels in the diet of dairy goats, which reflects that the rate of ruminal starch fermentation is higher in a high-RDS diet [46]. This result indicated that the rumen nitrogen utilization rate of AS could be improved by increasing the starch degradation rate when the effective availability of starch is the same.

In rumen, starch is degraded mainly by the α-amylases from rumen microorganisms to release maltose, glucose, and disaccharides, which are then converted into VFA and methane by glycolysis. In the present study, TVFA concentration was not affected by forage or grain. However, isobutyrate and valerate were higher for sheep fed AS. Similarly, Gsilon et al. [47] reported that feeding AS instead of AH (25% of diet DM) led to a decrease in isobutyrate and valerate in the rumen of dairy cows, probably because there were more free amino acids in AS, and more NH_3_-N, valerate, and isobutyrate were formed after microbial deamination [36]. Dietary urea supplementation has been shown to increase rumen butyric acid and isovalerate acid metabolism [48]. In the present study, the lactic acid content in the AH or LR diet was higher. This indicates that there has been increased propionic acid fermentation in the rumen, leading to the production of more propionic acid. Feeding AS to sheep significantly increased the height of the rumen papillae but decreased the width, which may be attributed to an increase in isobutyrate and valerate in rumen, which stimulates the growth of rumen papillae [49]; however, due to the insufficient physically effective fiber, the thickness of the stratum corneum of the rumen epithelium is reduced [50].

Our research further found that rumen total amino acids, isobutyrate, valerate, and lactic acid were negatively correlated with growth performance, and total amino acids and lactic acid were positively correlated with the F:G and dressing percentage, which further indicated that rumen amino acid would be absorbed and deposited into muscles through blood [40,41]. Isovalerate increased rumen weight and teat height but was negatively correlated with rumen weight and width [49].

The urea transporter-B (UT-B) and aquaporins (AQP3, 7, 10), which are localized in the epithelial layers of the ruminal papillae, are responsible for both the absorption and secretion of N [51]. The response of ruminal UT-B and AQP3 to dietary N intake has resulted in different findings in recent years. Under isoenergetic feeding conditions, UT-B expression appears to be unaffected by dietary N intake [52]. However, Ludden et al. [53] found that the expression of UT-B protein increased in lambs that were supplemented with RDP. Additionally, the UT-B and AQP7 mRNA were unaltered, while the AQP3 mRNA expression was also reduced in cattle supplemented with RDP [54]. It is not just related to the dietary N levels or degradability, and UT-B and AQP3 expression seemed to be affected by ruminal fermentable energy. Compared with hay-fed steer calves, grain diets had higher expressions of UT-B, AQP3 mRNA [55], and protein levels [56]. Similarly, a higher concentrate-to-forage ratio had a higher AQP3 mRNA in lambs [57] and upregulated UT-B mRNA and protein levels in goats [58]. In the current study, we observed that AQP3 and UT-B staining were mainly distributed in the stratum corneum, while AQP7 and AQP10 were distributed in all layers of the rumen epithelium. Furthermore, Zhong et al. [59] reported similar observations, with AQP3 and UT-B (25 kDa protein) staining in bovine rumen epithelium mainly observed in the stratum basale. Additionally, we also observed that AQP3 mRNA and protein levels were upregulated in the HR diet, regardless of whether sheep were fed AH or AS. AQP3 is a glycoprotein, and studies of the transporter usually detect both glycosylated and unglycosylated proteins [60]. For example, 42–45 and 25 kDa proteins found in the bovine rumen represent N-glycosylated (42–45 kDa) and nonglycosylated (25 kDa) forms of the AQP3 protein [59]. Similarly, in the current study, rumen protein AQP3 detected the 20 and 28–38 kDa bands and their expression patterns were similar. Therefore, we believe that the two distinct protein bands detected by our AQP3 antibody may represent N-glycosylated (28–38 kDa) and nonglycosylated (20 kDa) forms of the AQP3 protein [61]. This indicates that the long-term regulation of AQP3 proteins appears to be primarily influenced by dietary fermentable starch.

In the current study, the rumen microbiota richness (Chao index) was lower in sheep fed the HR diet, and the growth performance and feed efficiency of the HR diet were higher than those of the LR diet. It is reported that rumen microbial richness in rumen is negatively correlated with feed efficiency [62]. Similarly, Wang et al. [63] found a negative association between the ADG and Chao indices of rumen microbiota in goats.

Bacteria are the main participants in rumen feed degradation, and changes in diet composition usually affect the composition of rumen bacterial communities. The most dominant bacterial phyla in the rumen are *Bacteroidetes* and *Firmicutes*. In the current study, the composition of the rumen microorganism population was dominated by *Bacteroidetes* and *Firmicutes*. However, the phylum *Bacteroidetes* was more abundant in the AS diet than that in the AH. *Prevotella* was the predominant genus within *Bacteroidetes*, followed by *Rikenellaceae_RC9_gut_group* and *norank_f__F082* [27]. In the current study, the genera *Prevotella* was more abundant in the AS diet, which may be attributed to an increase in soluble fiber polysaccharides after ensilage fermentation, which increases the abundance of *Prevotella*. The anaerobic respiration of *Prevotella* produces acetate and isovalerate [64]. The significant increase in isobutyrate and valerate concentrations in AS also effectively demonstrates this point. Guo et al. [65] showed a higher abundance of *Bacteroidetes* and a lower abundance of *Firmicutes* in the rumen microbiome of corn silage inoculated with lignocellulose-degrading bacteria, with *Prevotella* being the most dominant genus and more abundant and with the concentration of VFA being significantly increased. *Rikenellaceae_RC9_gut_group* is associated with primary or secondary carbohydrate degradation and protein fermentation [66]. *norank_f__F082* is related to higher molar proportions of propionate [67]. In the present study, *Quinella* was the predominant genus within *Firmicutes*, followed by *Succiniclasticum*, *Christensenellaceae_R-7_group*, *Selenomonas,* and *UCG-004*. *Firmicutes* are known to be highly efficient at decomposing cellulose, and, therefore, their abundance positively correlates with carbohydrate fermentation in the rumen [68]. The abundance of *Firmicutes* in the ASLR group is lower than that in the other three groups, which may be the reason for the insufficient energy supply in rumen, but the difference among the four host species was not significant.

The metabolome results are consistent with previous findings [69,70] that showed that AS has lower rumen N efficiency than AH. The result that deserves to be highlighted is the distinct separation provided by the PCA, OPLS-DA, heatmap, and Venn diagrams of the different rumen fluid samples from sheep fed AH or AS, and there were some slight changes in grain sources. The HR diet enhanced sphingolipid metabolism and lysine degradation pathways while reducing aromatic amino acid biosynthesis. This is due to wheat containing 50% more lysine than corn [22]. Sphingolipids are important components of microbial cell membrane structure, and the high concentration of BCP in the HR diet enhances sphingolipid metabolism [71]. Starch is the most important nutrient in grain endosperm. Most starch can be degraded by the action of α-amylase into glucose, which is the precursor in the Embden–Meyerhof–Parnas (EMP) pathway [72]. The EMP pathway serves as the primary energy metabolism pathway of rumen bacteria under anaerobic conditions. We speculate that the starch in the HR diet stimulates microbial growth, and the resulting energy deficiency leads to the utilization of the microbial EMP pathway for glucose metabolism, which in turn decreases the biosynthesis of phenylalanine, tyrosine, and tryptophan biosynthesis. Moreover, our results found that compared with the AH diet, the AS diet only enhances the tryptophan metabolism pathways, while other pathways such as linoleic acid metabolism, nucleotide metabolism, purine metabolism, and amino acid biosynthesis were all downregulated in the rumen of sheep. Uracil and xanthine are nucleic acid breakup products resulting from the decomposition of rumen microbial bacteria and are often used as a biomarker for bacterial protein synthesis in the rumen. The increase in the levels of xanthine, uracil, alanine, and endotoxin in the rumen fluid of cows fed high-grain diets indicates widespread bacterial cell lysis [73]. These findings further suggested that silage fermentation results in alfalfa protein degradation, and the NPN from AS was not conducive to bacterial utilization (nucleotide metabolism and purine metabolism) [74]. Similarly, previous studies have shown that feeding more AS leads to inefficient N utilization and high urinary-N excretion [5]. However, the increasing RDS level in the diet only slightly improved the rumen purine metabolism of sheep fed AS, and it had little effect on amino acid synthesis. Our results support the previous theory that fermentations are more likely to be limited by the availability of carbohydrates or N than improved by protein and carbohydrate synchronization [12,75]. A previous study reported that increasing the proportion of high-moisture corn in the diet increased BCP yields to a greater extent for the cows fed the AS diet than for the cows fed the AH diet, but this study changed the dietary energy-to-nitrogen ratio [7]. After using the same nitrogen sources (pressed soybean meal and soybean meal), it was found that attempting to synchronize the degradability of ruminal non-structural carbohydrates and nitrogen produced minimal effects for mid-lactation dairy cows [16]. In addition to nitrogen metabolism, rumen linoleic acid and amino acid production are closely related to rumen bacteria [76]. Interestingly, linoleic acid metabolism was downregulated, while steroid hormone biosynthesis was enriched in the rumen of sheep fed AS. This indicates that alfalfa silage fermentation degrades fat into sterols, and there is less linoleic acid synthesis due to the lower bacterial activity in the rumen of AS sheep [40,77]. As expected, increasing the RDS level in the diet improved the synthesis of rumen linoleic acid, which may be the reason for enhancing rumen microbial activity.

Recent papers have reported that feeding bio-fermented rice straw to sheep, under the influence of the rumen microbiome, alters host phenotypes, including meat quality, methane production, and blood parameters [27]. In the current study, we found that *Selenomonadaceae, Quinella, and Christensenellaceae_R-7_group* were positively correlated with isobutyrate, valerate, NH_3_-N concentration, urase activity, tryptophan metabolism, and steroid hormone biosynthesis and negatively correlated with linoleic acid metabolism and amino acid biosynthesis. Those three bacterial genera belong to *Firmicutes* phyla and play an important role in nitrogen metabolism and fat degradation in alfalfa silage but do not participate in linoleic acid metabolism and amino acid biosynthesis [78]. The research also shows that dietary urea supplementation increased rumen ammonia nitrogen and *Christensenellaceae_R-7* in lambs [79]. Furthermore, correlation analysis also revealed that *norank_f_F082* was positively correlated with amino acid biosynthesis and negatively related to urase, which is consistent with the finding by [80]. *norank_f_F082* was related to higher molar proportions of propionate, and amino acids serve as precursors for propionate. Lysine, an essential AA involved in stimulating protein synthesis, has been reported to improve ADG, feed conversion, and N-balance in growing lambs when supplemented with rumen-protected lysine [81]. Lysine degradation products, including α-aminoadipic acid and 5-aminovaleric acid, have been reported to be consistent and repeatable biomarkers of residual feed intake [82]. Similar findings were found in the correlation analysis of this study; 5-aminopentanoic acid, a product of lysine catabolism, has a positive correlation with ADG, which may be the reason for the higher growth performance of sheep fed with HR diets. As expected, BCP was positively correlated with the linoleic acid metabolic pathways (9-Hpode and 13-L-Hydroperoxylinoleic acid) and was negatively associated with the steroid synthesis pathway (3a, 21-Dihydroxy-5B-Pregnane-11,20-dione). This indicates that increasing the population of rumen bacteria can promote the biosynthesis of linoleic acid [76].

## 5. Conclusions

Increased RDS in the diet improved the growth performance and rumen N utilization and reduced bacterial diversity in sheep. The alfalfa silage diet only increased feed efficiency; it did not affect growth performance. Additionally, it decreased rumen nitrogen utilization, linoleic acid, and amino acid biosynthesis. Nevertheless, there were limited interactions between forage and RDS; increased RDS in the AS diet enhanced the nitrogen capture rate of rumen microorganisms for alfalfa silage, with only slight improvements in the purine metabolism, linoleic acid, and amino acid synthesis.

## Figures and Tables

**Table 1:** Ingredient compositions and nutritive values of experimental feeds (dry matter basis).

Ingredients	Treament ^1^				Nutrient	Treament			
	AHLR	AHHR	ASLR	ASHR		AHLR	AHHR	ASLR	ASHR
Corn stalk	10	10	10	10	Dry matter	87.75	86.28	66.03	64.56
Alfalfa hay	40	40	0	0	Digestible energy, MJ/kg	12.18	12.18	12.14	12.14
Alfalfa silage	0	0	40	40	Crude protein	13.93	13.84	14.33	14.24
Corn	39	10	39	10	starch	28.15	28.40	29.87	30.12
wheat	0	33	0	33	Acid detergent fiber	20.57	20.37	20.33	20.13
Soybean meal	5	1	5	1	Neutral detergent fiber	30.50	30.60	29.34	29.44
Wheat bran	3.75	2.75	3.75	2.75	Ether extract	3.72	3.71	4.12	4.11
Soybean oil	0.25	1.25	0.25	1.25	RDS ^3^	14.85	20.21	14.85	20.21
Calcium hydrogen Phosohate	0.25	0.25	0.25	0.25	RDP ^4^	2.94	2.94	4.38	4.38
limestone	0.75	0.75	0.75	0.75	RDS/RDP	5.05	6.87	3.39	4.61
Salt	0.5	0.5	0.5	0.5					
Premix ^2^	0.5	0.5	0.5	0.5					

^1^ AHLR: alfalfa hay and low RDS, AHHR: alfalfa hay and high RDS, ASLR: alfalfa silage and low RDS, ASHR: alfalfa silage and high RDS. ^2^ The premix contained/kg diet: vitamin A 6000 IU, vitamin D3 2000 IU, vitamin E 15 IU, vitamin K3 1.8 mg, vitamin B1 0.35 mg, vitamin B2 8.5 mg, vitamin B6 0.9 mg, vitamin B12 0.03 mg, D-pantothenic acid 16 mg, nicotinic acid 22 mg, folic acid 1.5 mg, biotin 0.15 mg, Cu 8 g, Fe 40 mg, Mn 20 mg, Zn 40 mg, I 0.8 mg, Se 0.3 mg, Co 0.3 mg. ^3^ RDS: rumen-degradable starch. ^4^ RDP: rumen-degradable protein.

**Table 2:** Effect of rumen-degradable starch on growth performance, dressing percentage, and stomach weight in sheep fed alfalfa hay or silage.

Items	Treatments ^1^				SEM	*p*-Value ^2^		
	AHLR	AHHR	ASLR	ASHR		Forage	RDS	Forage × RDS
Dry matter intake, g/d	1039.06	1121.17	980.34	1072.27	43.51	0.205	0.045	0.907
Average daily gain, g/d	156.12 ^b^	195.88 ^a^	166.66 ^ab^	203.14 ^a^	15.03	0.217	0.003	0.565
F:G	6.99 ^a^	6.06 ^ab^	6.11 ^ab^	5.29 ^b^	0.44	0.062	0.048	0.892
Live weight, kg	36.30	38.75	36.51	38.82	1.4	0.879	0.115	0.963
Hot carcass, kg	17.28	18.18	17.14	17.68	0.37	0.710	0.368	0.815
Dressing percentage, %	47.58	46.61	47.07	45.43	0.01	0.155	0.070	0.522
Stomach weight, g								
Rumen	575.59	645.84	548.51	661.76	41.69	0.894	0.033	0.609
Reticulum	102.3	91.2	115.71	104.21	7.94	0.110	0.168	0.980
Omasum	127.41	135	126.36	127.56	14.72	0.793	0.786	0.844
Abomasal	92.00	134.59	127.95	117.26	13.18	0.481	0.285	0.051
Total stomach weight	897.30	1006.63	918.54	1010.80	61.47	0.845	0.130	0.895

^1^ AHLR: alfalfa hay and low RDS, AHHR: alfalfa hay and high RDS, ASLR: alfalfa silage and low RDS, ASHR: alfalfa silage and high RDS. ^2^ Forage = alfalfa hay versus alfalfa silage (AH vs. AS); RDS = low rumen-degradable starch versus high rumen-degradable starch (LR vs. HR); forage × RDS, forage by RDS interaction. ^a,b^ Significant differences within a row with different superscripts (*p* < 0.05).

**Table 3:** Effect of rumen-degradable starch on rumen fermentation parameters and rumen papillae morphology in sheep fed AH or AS.

Items ^1^	Treatments ^2^				SEM	*p*-Value ^3^		
	AHLR	AHHR	ASLR	ASHR		Forage	RDS	Forage × RDS
pH	5.9	5.8	5.7	5.7	0.06	0.215	0.736	0.736
NH_3_-N, mg/100 mL	7.66 ^c^	8.37 ^bc^	15.39 ^a^	10.53 ^b^	0.66	0.001	0.010	0.002
TAA, μmol/mL	16.67 ^a^	13.53 ^b^	18.03 ^a^	16.19 ^a^	0.52	0.126	0.032	0.678
BCP, mg/mL	33.46 ^b^	54.24 ^a^	28.01 ^b^	30.85 ^b^	2.71	0.003	0.009	0.055
α-amylase activity, U/dL	11.68	11.87	7.55	12.49	1.51	0.279	0.097	0.111
Cellulase activity, U/min/mL	33.22	28.32	23.82	28.82	4.71	0.420	0.939	0.333
Urase activity, U/min/mL	15.54	20.56	26.95	23.04	1.45	0.041	0.910	0.189
VFA, mmol/L								
Acetate	70.31	71.81	80.25	77.58	5.12	0.139	0.910	0.690
Propionate	29.54	27.55	28.41	25.01	3.53	0.621	0.467	0.849
Isobutyrate	3.01 ^c^	3.18 ^bc^	4.36 ^a^	4.01 ^ab^	0.37	0.002	0.794	0.424
Butyrate	3.47	3.28	3.92	4.18	0.43	0.133	0.929	0.617
Isovalerate	2.17	2.29	2.96	2.42	0.34	0.187	0.535	0.342
Valerate	0.93	0.93	1.16	1.27	0.13	0.035	0.681	0.674
TVFA	109.43	109.04	121.05	114.47	8.37	0.331	0.689	0.722
Lactic acid	0.53 ^a^	0.39 ^bc^	0.46 ^ab^	0.32^c^	0.04	0.029	0.001	0.905
A/P	2.55	2.89	2.91	3.46	0.32	0.145	0.161	0.745
Rumen papillae morphology, μm								
Papillae height	2206.69	2321.88	2506.08	2520.3	129.8	0.082	0.641	0.716
Papillae width	430.12 ^a^	412.6 ^a^	346.67 ^b^	376.68 ^ab^	23.28	0.009	0.771	0.273
Papillae surface area, μm^2^	94.48	97.63	83.75	93.88	6.88	0.344	0.385	0.646
Lamina propria thickness	651.69	658.02	571.69	734.82	65.37	0.981	0.205	0.239
Muscle layer thickness	1599.85	1533.79	1717.24	1752.04	155.99	0.305	0.923	0.756

^1^ TAA: total amino acid; BCP: bacterial protein. ^2^ AHLR: alfalfa hay and low RDS, AHHR: alfalfa hay and high RDS, ASLR: alfalfa silage and low RDS, ASHR: alfalfa silage and high RDS. ^3^ Forage = alfalfa hay versus alfalfa silage (AH vs. AS); RDS = low rumen-degradable starch versus high rumen-degradable starch (LR vs. HR); forage × RDS, forage by RDS interaction. ^a–c^ Significant differences within a row with different superscripts (*p* < 0.05).

## Data Availability

The data presented in this study are available upon request from the corresponding author.

## Supplementary Materials

The following supporting information can be downloaded at https://www.mdpi.com/2076-2615/15/1/34/s1, Supplementary File S1: In vitro experiment. Supplementary File S2: Table S1: Chemical compositions of forage and grain; Table S2: Primer sequences used for quantitative real-time PCR analysis; Figure S1: The complete picture of Western blotting. Supplementary File S3: Table S3: Differential metabolite information (AHLR_VS_AHHR). Table S4: Differential metabolite information (AHLR_VS_ASLR); Table S5: Differential metabolite information (AHLR_VS_ASHR); Table S6: Differential metabolic pathway heat map.

## Abbreviations

AA	Amino acid
ADG	Average daily gain
AQP3	Aquaporins 3
AQP7	Aquaporins 7
AQP10	Aquaporins 10
AH	Alfalfa hay
AS	Alfalfa silage
AHLR	Alfalfa hay and low rumen-degradable starch group
AHHR	Alfalfa hay and high rumen-degradable starch group
ASLR	Alfalfa silage and low rumen-degradable starch group
ASHR	Alfalfa silage and high rumen-degradable starch group
BCP	Bacterial protein
DM	Dry matter
DMI	Dry matter intake
F:G	Feed to gain
OM	Organic matter
RDP	Rumen-degradable protein
RDS	Rumen-degradable starch
N	Nitrogen
NH_3_-N	Ammonia-N
NPN	Non-protein nitrogen
UT-B	Urea transporter-B
TAA	total amino acids
TVFA	Total volatile fatty acid
VFA	Volatile fatty acid
