# Supplementary material for: Rumen-Degradable Starch Improves Rumen Fermentation, Function, and Growth Performance by Altering Bacteria and Its Metabolome in Sheep Fed Alfalfa Hay or Silage
Source: Animals (Basel). 2024 Dec 26;15(1):34. doi: 10.3390/ani15010034 (PMC11870059; doi:10.3390/ani15010034)
Supplement: Supplementary file 1 [file animals-15-00034-s001.zip › Additional file S1.pdf]

Additional file S1 In vitro experiment

Effects of different proportions of wheat and corn in substrate with alfalfa silage on in vitro rumen fermentation and microbial protein synthesis

Abstract: Alfalfa silage has complete protein content, because its high rumen degradable protein proportion will increase Urea-N output, and increasing dietary rumen degradable starch can enhance ruminal microbial protein synthesis. This study was conducted to investigate the influence of different proportion of wheat and corn in substrate with alfalfa silage on in vitro rumen fermentation and nitrogen (N) utilization. The single factor design included five replacement levels of wheat to corn in substrate with alfalfa silage (0:100, 25:75, 55:45, 77:23, 100:0), and each substrate was incubated for 3, 6, 12, and 24 h, then, the pH, α-amylase, ammonia nitrogen (NH_3-N), bacterial protein (BCP), production of gases, and volatile fatty acids were measured. The total gas production linearly increased with increasing level of wheat from 3 h to 10 h of incubation (P<0.001), and no difference was observed between five groups after 11h of incubation. On h 3 of incubation, the pH and NH_3-N reduced quadratically as the wheat starch level increased, however, the BCP and α-amylase increased linearly or quadratically as the wheat level increased (P<0.05). On h 6 of incubation, the NH_3-N reduced linearly or quadratically as the wheat starch level increased, and the acetate, propionate and TVFA concentrations increased linearly as the wheat level increased in substrates (P<0.001). On h 12 of incubation, the BCP and α-amylase increased linearly or quadratically as the wheat level increased (P<0.05). The propionate concentration increased linearly as the wheat level in substrate increased on h 12 of incubation (P<0.05). On h 24 of incubation, the α-amylase increased linearly as the wheat level increased (P<0.05). The highest multiple-factor associative effects index was observed in a ratio of 77:23 substrate, therefore, the proportion of wheat to corn was 77: 23 in the diet resulted in a desirable rumen N utilization.

1. Introduction

We hypothesized that increasing starch rumen degradability of finishing diets will improve the nitrogen utilization rate of alfalfa silage and stimulate synthesis of microsomal proteins in vitro rumen. Thus, an experiment was designed to study the effects of different proportions of wheat to corn (0:100, 25:75, 55:45, 77:23 and 100:0) in substrate with alfalfa silage on in vitro rumen fermentation and microbial protein synthesis.

2. Materials and Methods

2.1 Composition and Nutritional Level of Experimental Diet

The alfalfa was collected on the Hohhot, Inner Mongolia, China. The second cut of pure alfalfa plants (cultivar WL354) were harvested at the early bud stage of maturity (geometric mean cut length of 2.0 ± 0.1 cm) and equally spread on a white plastic sheet in the shade to low-intensity wilting treatment until DM concentrations of 400 g/kg were reached, then packed into the white plastic sheet and vacuum sealed and stored. Corn, wheat, corn stalk and other ingredients were obtained from a local feed mill. All ingredients were then dried in a forced-air oven at 65 ◦C for 48 h and ground in a mill to pass through a 1-mm sieve for later analysis, the samples dry matter (DM, 105 °C), crude protein (CP), ether extract (EE), ash, acid detergent fiber (ADF), and neutral detergent fiber (NDF) were analyzed. The starch was determined with commercial kits (Nanjing Jiancheng Institute of Bioengineering, Nanjing, China). Five kinds of substrates used in this study were prepared according to the proportion of wheat starch to corn starch in the diet and the chemical composition and nutritional level of the every substrates are listed in Table 1.

Table 1. Composition and nutritional level of substrate in vitro experiment(air-dry basis)

Ingredients	Proportion of wheat to corn in the substrate[1]					Nutrient	Proportion of wheat to corn in the substrate				
	0：100	25：75	55：45	77：23	100：0		0：100	25：75	55：45	77：23	100：0
Corn stalk	10	10	10	10	10	Dry matter	66.74	66.13	65.43	65.27	65.12
Alfalfa silage	40	40	40	40	40	Digestible energy	2.95	2.93	2.91	2.91	2.90
Wheat	0	10	23	33	43.5	Crude protein	14.64	14.67	14.45	14.56	14.73
Corn	39	30	19	10	0	starch	28.55	28.47	28.85	28.80	28.37
Soybean meal	5	4	2	1	0	Acid detergent fiber	21.27	21.20	21.08	21.07	21.12

Wheat bran	4	3.5	3	3	3.5	Neutral detergent fiber	30.22	30.19	30.14	30.32	30.65
Soybean oil	0	0.5	1	1	1	Ether extract	4.05	4.25	4.30	4.04	3.78
Calcium Hydrogen Phosohate	0.25	0.25	0.25	0.25	0.25	RDS[2]	14.85	16.40	18.67	20.21	21.62
limestone	0.75	0.75	0.75	0.75	0.75	RDP[3]	6.32	6.32	6.32	6.32	6.32
Salt	0.5	0.5	0.5	0.5	0.5	RDS/RDP	2.35	2.60	2.95	3.20	3.47
Premix[1]	0.5	0.5	0.5	0.5	0.5						

[1] The premix contained/kg diet: vitamin A 6 000 IU, vitamin D_3 2000 IU, vitamin E 15 IU, vitamin K_3 1.8 mg, vitamin B_1 0.35 mg, vitamin B_2 8.5 mg, vitamin B_6 0.9 mg, vitamin B_{12} 0.03 mg, *D*-pantothenic acid 16 mg, nicotinic acid 22 mg, folic acid 1.5 mg, biotin 0.15 mg, Cu 8 g, Fe 40 mg, Mn 20 mg, Zn 40 mg, I 0.8 mg, Se 0.3 mg, Co 0.3 mg.

[2] RDS: Rumen degradable starch

[3] RDP: Rumen degradable protein

2.2 In vitro experiment

Rumen fluid was obtained from six Hu-sheep (2.5 years old, body weight of 42±3kg) housed at the experimental farm located in Hohhot. Each sheep fed *ad libitum* a total mixed ration containing (per kg DM) corn stalk (340g), alfalfa silage (260g), alfalfa hay (200g) and concentrate (200g). Rumen fluid was collected before the morning feeding using a tube attached to a vacuum pump and strained through 4 layers of gauze into a pre-warmed flasks with 39°C and full of carbon dioxide, sealed the top and taken to the laboratory.

The five substrates with different proportions of wheat to corn (0:100, WTC0; 25:75,WTC25; 55:45, WTC55; 77:23,WTC77; and 100:0,WTC100) were incubated in triplicate for 3, 6, 12, and 24 h,respectively, and a real-time monitoring for gas production was conducted, resulting in a total of 75 bottles used for incubation (i.e., 5 WTC × 4 sampling times × 3 replicates + 5 WTC × 1 gas production device × 3 replicates). The rumen fluid was mixed with a reduced and pre-warmed buffer at a ratio of 1:2 under continuous CO_2 flushing[18]. After equilibrating the solution with CO_2, 60 mL aliquot of rumen fluid–buffer mixture was added to 100-mL incubation bottles containing 1.0 g of the substrate. Bottles were closed with a butyl rubber stopper, and randomly placed in a constant temperature oscillation incubator (120 r/min) at 39°C. In the 75 bottles, 15 bottles (5 WTC× 3 replicates) were connected to the gas measurement system (ANKOM RFS) via a needle, and gas production were recorded automatically every minute, a total of 75 incubation bottles were carried out simultaneously. All above processes were anaerobic. The samples were collected on h 3, 6, 12, and 24 of incubation from 15 bottles (5 WTC× 3 replicates) removed out from the incubator and placed in ice water to stop fermentation, and then pH was measured. The mixed fluid was then transferred into micro-centrifuge tube and centrifuged (3000r/min-1) to separate the upper clear fluid, and stored under -20°C used for measuring volatile fatty acids (VFA), including acetate, propionate, butyrate, isbutyric, valerate and

isovalerate. The VFA content was analyzed by gas chromatography (6890N; Agilent, California, United States) . The ammonia nitrogen (NH_3-N) and bacterial protein (BCP) content were assessed by spectrometer using colorimetry [19] and coomath bright blue process, respectively. The gas production was calculation. The α-amylase was determined with commercial kits (Nanjing Jiancheng Institute of Bioengineering, Nanjing, China). The pH was measured using a portable pH meter. Single-factor associative effects index (SFAEI) and multiple-factor associative effects index (MFAEI) were calculated according to the equations (1) and (2) .

SFAEI=Σnm=1(A2m−A1m)/n/A2m (1)

Not: m is each incubation time (i.e., m=1, 2, 3 and 4); n is the number of incubation time (n=4); A1m is the value of each single index in the control group (WTC0) at different sampling times. A2m is the value of each single index in the experimental group (WTC 25, WTC55, WTC77 and WTC100) at different culture time.

MFAEI = the sum of each single − factor associative effect value (2)

Not: In our study, MFAEI is the sum of the four SFAEI of TVFA, α-amylase, GP and MCP.

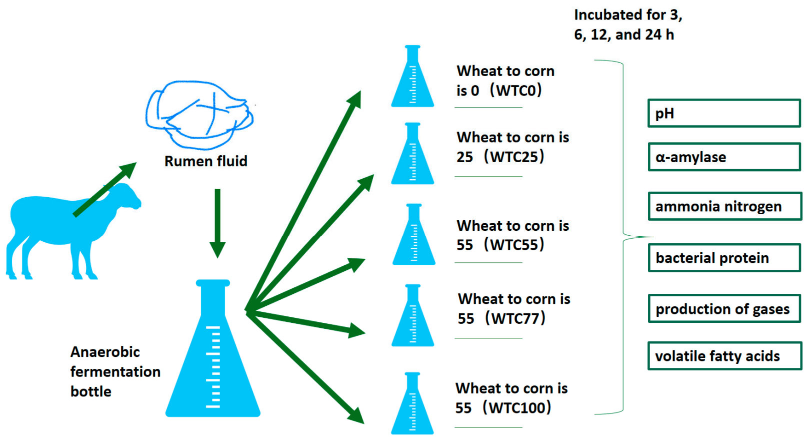

2.3 Statistical Analysis

The data were analyzed using the General Linear Model (GLM) procedure of SAS (Version 9.2, SAS Institute Inc.). Post hoc multiple comparison between treatments was by Duncan's multiple range test (DMRT), and considered significant at $p < 0.05$, extremely significant at $p < 0.01$, data are presented as mean ± SD.

3. Results

3.1. Effects of different proportion of wheat to corn in substrate with alfalfa silage on total gas production of the in vitro rumen fermentation

The total gas production of the in vitro rumen fermentation of the different substrates were shown in Figure 1. The total gas production had no significant difference among groups at h 2, but linearly increased with increasing level of wheat from h 3 to 10 of incubation (P<0.01), however, no difference was observed between five groups from h 11 to 25 of incubation.

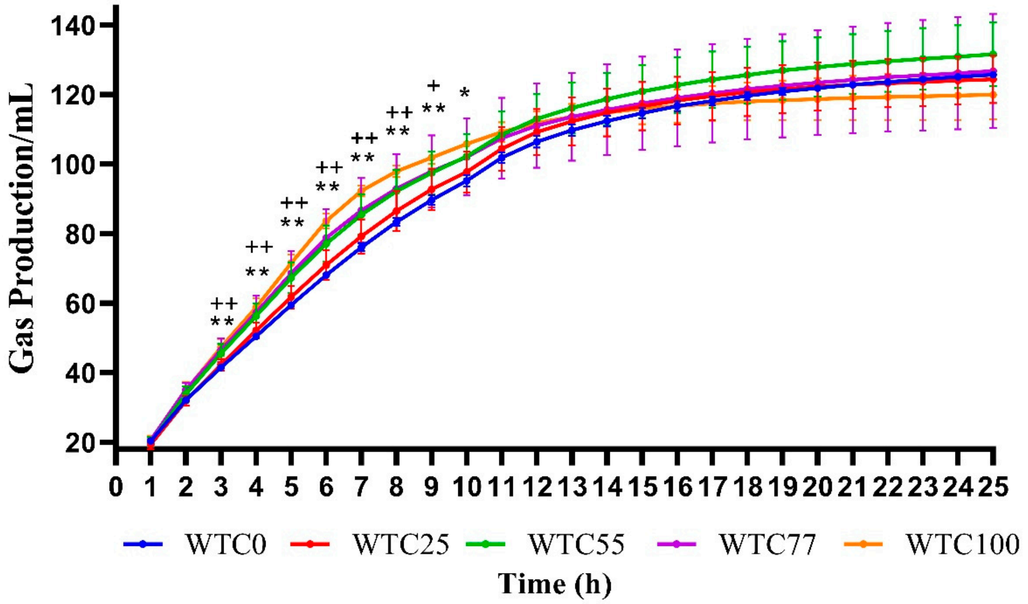

Figure 1. Gas production from the in vitro rumen incubation of different proportions of wheat to corn in substrate with alfalfa silage. Data presented as real-time measurement of total gas produced as mL/h of

different group throughout a 24-h incubation. Three biological replicates of each treatment in vitro incubations. * means linear: P<0.05, ** means linear: P<0.01, + means quadratic: P<0.05, ++ means quadratic: P<0.01.

3.2. Effects of different proportion of wheat to corn in substrate with alfalfa silage on in vitro rumen fermentation indexes

The effects of different proportion of wheat to corn in substrate with alfalfa silage on the in vitro rumen fermentation indexes were shown in Table 2. At h 3 of incubation, the pH reduced linearly as the wheat level increased (P<0.05), the NH_3-N reduced quadratically as the wheat level increased (P<0.05). On the contrary, the BCP and α-amylase increased linearly or quadratically as the wheat level increased(P<0.01). At h 6 of incubation, the NH_3-N reduced linearly or quadratically as the wheat level increased (P<0.001). At h 12 of incubation, the BCP and α-amylase increased linearly or quadratically as the wheat level increased (P<0.05). At h 24 of incubation, the α-amylase increased linearly as the wheat levels increased (P<0.05). No significant difference was found in the content of other variables.

Table 2. Effects of different proportion of wheat to corn in substrate with alfalfa silage on in vitro rumen fermentation

Itmes	Proportion of wheat to corn in the substrate					SEM	*P*-values[a]	
	WTC0	WTC25	WTC55	WTC77	WTC100		L	Q
3h								
pH	6.67	6.65	6.65	6.63	6.64	0.01	0.035	0.101
NH_3-N, mg/100ml	21.83	21.94	20.64	20.38	21.44	0.37	0.106	0.035
BCP, mg/100ml	20.09	19.49	21.13	23.37	22.06	0.69	0.001	0.001

α-amylase, U/dL	272.39	302.15	319.89	317.60	343.35	26.6	0.001	0.001
6h								
pH	6.46	6.40	6.41	6.37	6.34	0.03	0.063	0.191
NH_3-N, mg/100ml	23.21	23.00	22.36	20.92	21.78	0.44	0.001	0.001
BCP, mg/100ml	22.12	21.60	22.28	23.30	22.62	0.64	0.182	0.415
α-amylase, U/dL	203.72	235.77	247.21	276.39	244.35	37.08	0.094	0.109
12h								
pH	6.18	6.14	6.13	6.09	6.09	0.03	0.099	0.263
NH_3-N, mg/100ml	21.82	21.82	21.30	21.78	21.40	0.67	0.065	0.185
BCP, mg/100ml	25.78	25.68	26.15	25.90	27.22	0.37	0.007	0.005
α-amylase, U/dL	267.83	275.42	304.01	291.17	286.51	15.87	0.045	0.012
24h								
pH	6.04	6.00	5.98	5.94	6.01	0.04	0.496	0.555
NH_3-N, mg/100ml	26.50	26.47	25.75	26.17	27.39	0.45	0.525	0.158
BCP, mg/100ml	23.26	23.85	23.33	23.24	23.00	0.37	0.343	0.412
α-amylase, U/dL	187.89	179.14	195.48	240.41	211.82	30.38	0.043	0.141

[a] L, linear; Q, Quadratic, same as below.

Effect of different proportion of wheat to corn in substrate with alfalfa silage on in vitro rumen fermentation volatile fatty acid yield shown in table 3. The acetate, propionate and TVFA concentrations increased linearly or quadratically as the wheat levels increased up were in substrates after 6 h of incubation ($P<0.001$). The propionate concentrations increased linearly as the wheat levels increased up were in

substrates after 12 h of incubation (P<0.05), whereas after 24 h of incubation, no differences in volatile fatty acid yield were observed.

Table 3. Effect of different proportion of wheat to corn in substrate with alfalfa silage on in vitro rumen fermentation volatile fatty acid yield (mmol/L)

Items	Proportion of wheat to corn in the substrate					SEM	*P*-values	
	WTC0	WTC25	WTC55	WTC77	WTC100		L	Q
3h								
Acetate	12.47	12.98	12.99	13.36	12.88	0.83	0.655	0.815
Propionate	4.74	5.07	5.17	5.16	5.24	0.31	0.264	0.488
Isoacids	2.29	2.20	2.10	2.15	2.00	0.25	0.445	0.756
Butyrate	2.53	2.47	2.36	2.38	2.17	0.23	0.278	0.556
isovaleric acid	4.27	3.75	4.02	4.19	3.81	0.43	0.753	0.947
valerianic	2.05	2.04	1.91	1.94	1.77	0.19	0.279	0.557
TVFA	28.35	28.51	28.54	29.18	27.87	1.96	0.975	0.960
A/P	2.66	2.56	2.51	2.59	2.44	0.10	0.206	0.464
6h								
Acetate	14.63	15.21	15.55	16.69	17.21	0.68	0.001	0.001
Propionate	5.62	5.80	6.05	6.48	7.00	0.33	0.001	0.001
Isoacids	2.71	2.73	2.69	2.57	2.55	0.11	0.197	0.406
Butyrate	2.96	3.01	3.02	3.07	3.07	0.14	0.547	0.837

isovaleric acid	4.47	4.53	4.46	4.52	4.60	0.18	0.678	0.897
valerianic	3.35	3.22	3.25	3.26	3.04	0.11	0.121	0.270
TVFA	33.73	34.50	35.02	36.59	37.47	1.14	0.003	0.014
A/P	2.60	2.62	2.57	2.58	2.46	0.08	0.188	0.311
12h								
Acetate	23.26	24.87	24.62	24.24	25.88	1.03	0.170	0.405
Propionate	9.05	9.79	10.26	9.97	10.24	0.39	0.029	0.448
Isoacids	3.33	3.23	3.29	3.43	2.96	0.27	0.562	0.697
Butyrate	4.69	4.47	5.19	5.00	5.05	0.27	0.135	0.328
isovaleric acid	6.10	6.00	6.25	5.64	5.64	0.25	0.118	0.219
valerianic	6.54	6.42	7.38	6.87	6.44	0.33	0.723	0.245
TVFA	52.98	54.77	56.98	55.14	56.20	1.34	0.096	0.139
A/P	2.57	2.54	2.40	2.43	2.53	0.07	0.392	0.250
24h								
Acetate	27.02	27.60	27.46	28.09	29.16	1.31	0.276	0.526
Propionate	9.65	10.22	10.32	10.75	11.09	0.56	0.053	0.165
Isoacids	3.82	4.23	4.30	4.62	4.94	0.53	0.121	0.314
Butyrate	5.80	5.63	5.83	6.66	6.79	0.59	0.246	0.116
isovaleric acid	7.53	7.39	7.15	7.08	8.50	0.84	0.591	0.518
valerianic	7.34	7.44	7.94	7.64	8.97	0.82	0.201	0.387
TVFA	61.16	62.51	63.00	64.83	69.45	4.02	0.151	0.314

A/P	2.80	2.69	2.66	2.61	2.66	0.10	0.296	0.461

3.3 Effects of different proportion of wheat to corn in substrate with alfalfa silage on in vitro rumen fermentation SFAEI and MFAEI

The SFAEI of TVFA, α-amylase, GP and BCP of different proportion of wheat to corn in substrate with alfalfa silage on the in vitro rumen fermentation were shown in Figure 2. The SFAEI of TVFA, α-amylase, GP and BCP increased linearly as the wheat levels increased up, and the highest BCP, GP, α-amylase and TVFA were in WTC77, WTC55, WTC77 and WTC100 substrate, respectively. the highest MFAEI was in WTC77 substrate.

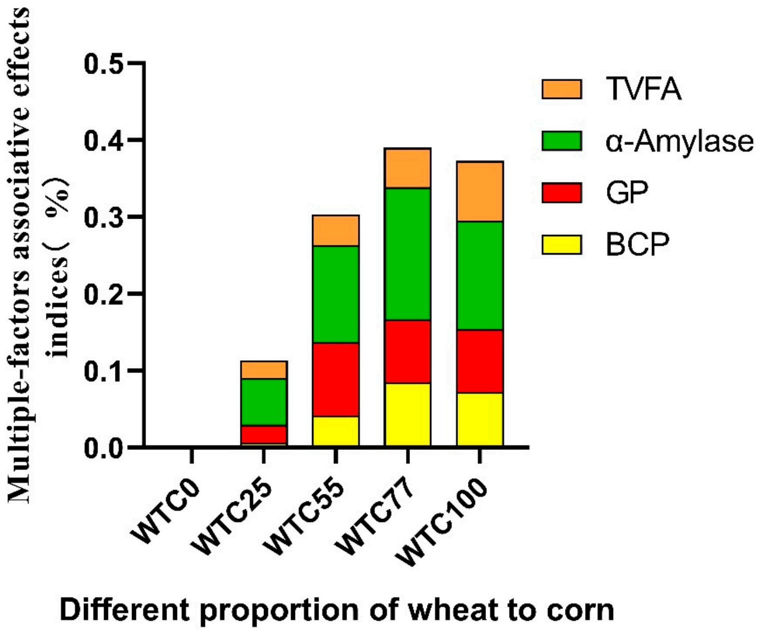

Figure 2. Single-factor associative effects indices (SFAEI) and multiple-factors associative effects indices (MFAEI) of different proportions of wheat to corn in substrate with alfalfa silage in vitro rumen incubation. TVFA, total volatile fatty acid; GP, gas production; BCP, bacterial protein.

4. Conclusions

In the current study, the total gas production, BCP, α-amylase and TVFA increased with increasing level of wheat from h 3 to 12 of incubation, and NH_3-N and pH reduced linearly, and the highest BCP content and MFAEI were in WTC77 substrate. Therefore, the proportion of wheat to corn was 77:23 in the diet results in a desirable rumen N utilization.
