# Supplementary material for: Rumen-Degradable Starch Improves Rumen Fermentation, Function, and Growth Performance by Altering Bacteria and Its Metabolome in Sheep Fed Alfalfa Hay or Silage
Source: Animals (Basel). 2024 Dec 26;15(1):34. doi: 10.3390/ani15010034 (PMC11870059; doi:10.3390/ani15010034)
Supplement: Supplementary file 1 [file animals-15-00034-s001.zip › Additional file S2.pdf]

Additional file S2

Table S1 Chemical compositions of Forage and Grain

Nutrient	Forage		Grain	
	Alfalfa hay	Alfalfa silage	Corn	Wheat
Dry matter	91.70	37.40	86.90	85.70
Digestible energy	2.62	2.57	3.15	3.45
Crude protein	18.30	19.10	8.50	13.50
starch	1.50	4.80	70.40	63.10
Acid detergent fiber	30.75	26.58	3.60	4.20
Neutral detergent fiber	46.67	36.67	9.80	12.50
Ether extract	2.50	3.50	3.84	1.98
RDS[2]	-	-	38.08	49.71
RDP[3]	43.00	58.00	-	-

Table S2 Primers sequences used for quantitative real-time PCR analysis.

**Gene**	**Gene Bank NO.**	**Primer Sequence**	**Fragment Size**	**Annealing temp (°C)**
GADPH	NC_056056.1	F- GGGTCATCATCTCTGCACCT<br>R- GGTCATAAGTCCCTCCACGA	122	60
AQP3	NC_056055.1	F- CTTCGGGTTGTATTACGATGCG<br>R-CCAAGTGTCCAGAAGGGTAGGTG	118	56
AQP7	NC_056055.1	F- GCCACCATCTACAGCCTCTTC<br>R- GTCATGTGGTCAGGAAGGTAGGT	119	61
AQP10	NC_056054.1	F- TAGCCACCTATGTGAGTGGTAATG<br>R- CACCAGGGAGTAAATGAGAAACCT	116	61
UT-B	NC_056076.1	F- AATCAGGCTCCACAAGGTCAAG<br>R- CCGAAGAACCCAGTCAACGA	161	65

Fig S3. The complete picture of western blotting

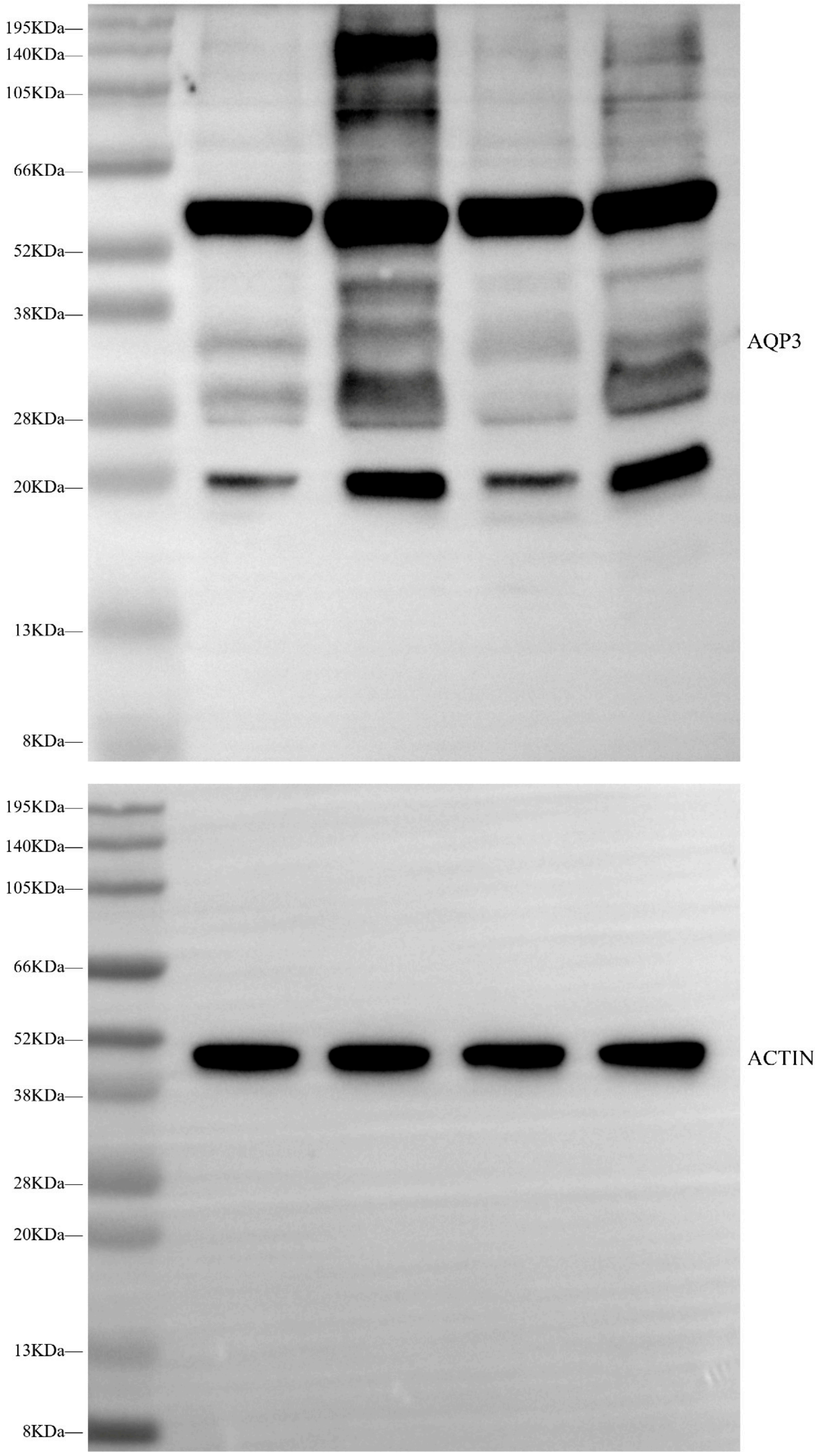
195KDa
140KDa
105KDa
66KDa
52KDa
38KDa
28KDa
20KDa
13KDa
8KDa
AQP3
195KDa
140KDa
105KDa
66KDa
52KDa
38KDa
28KDa
20KDa
13KDa
8KDa
ACTIN

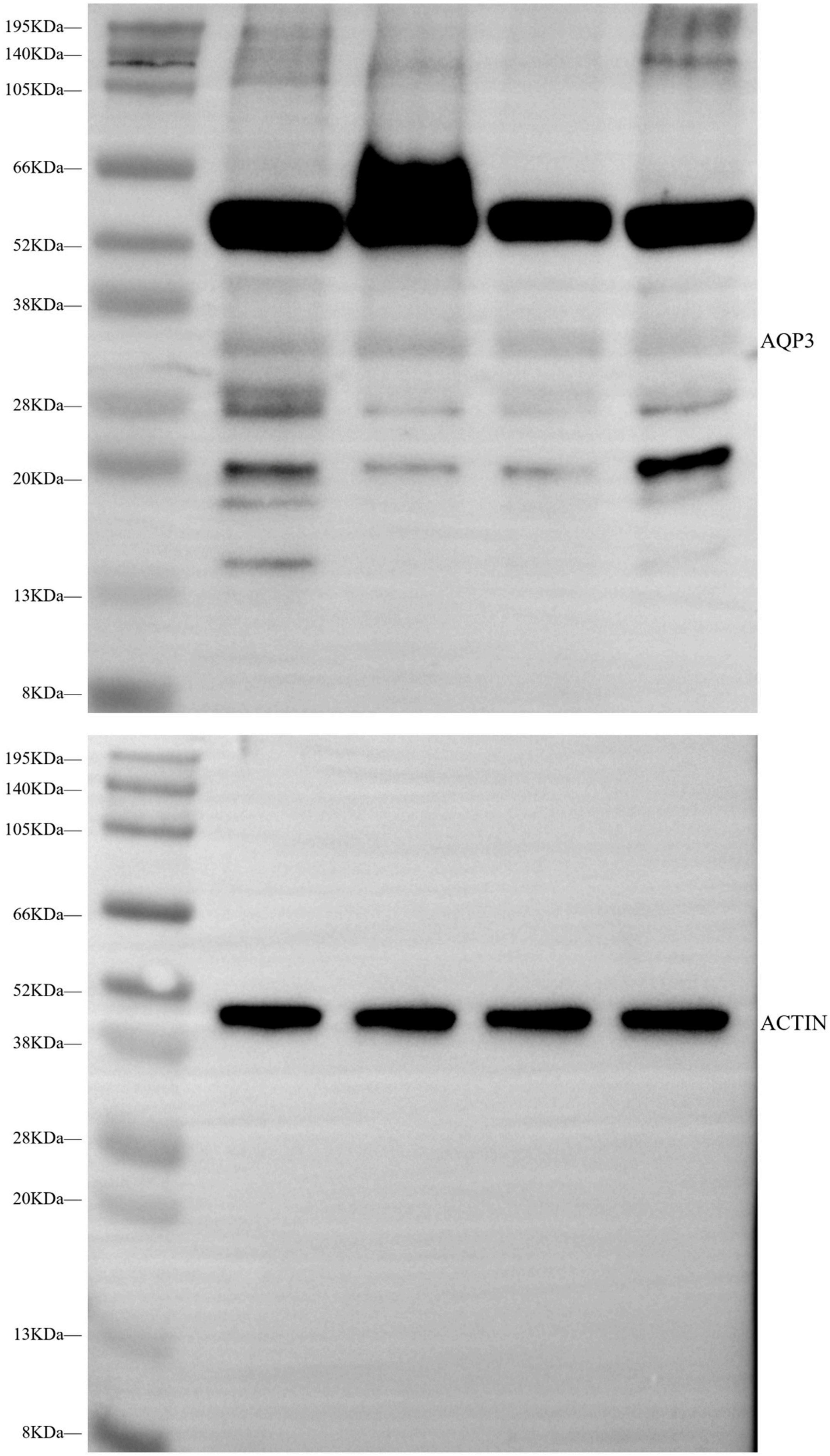
195KDa
140KDa
105KDa
66KDa
52KDa
38KDa
28KDa
20KDa
13KDa
8KDa
AQP3
195KDa
140KDa
105KDa
66KDa
52KDa
38KDa
28KDa
20KDa
13KDa
8KDa
ACTIN

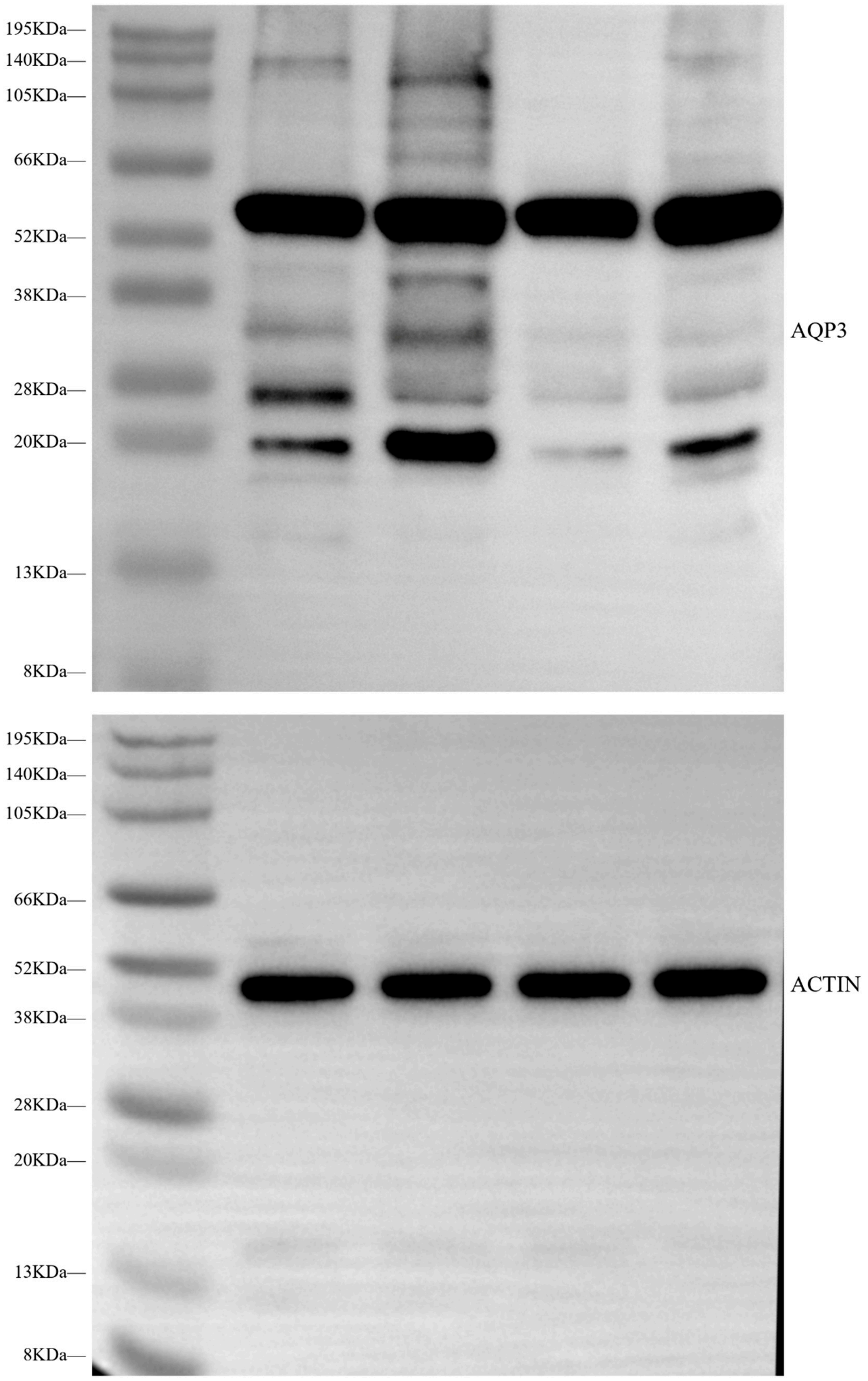
195KDa
140KDa
105KDa
66KDa
52KDa
38KDa
28KDa
20KDa
13KDa
8KDa
AQP3
195KDa
140KDa
105KDa
66KDa
52KDa
38KDa
28KDa
20KDa
13KDa
8KDa
ACTIN
