# Supplementary figures and images for: Rumen-Degradable Starch Improves Rumen Fermentation, Function, and Growth Performance by Altering Bacteria and Its Metabolome in Sheep Fed Alfalfa Hay or Silage
Source: Animals (Basel). 2024 Dec 26;15(1):34. doi: 10.3390/ani15010034 (PMC11870059; doi:10.3390/ani15010034)

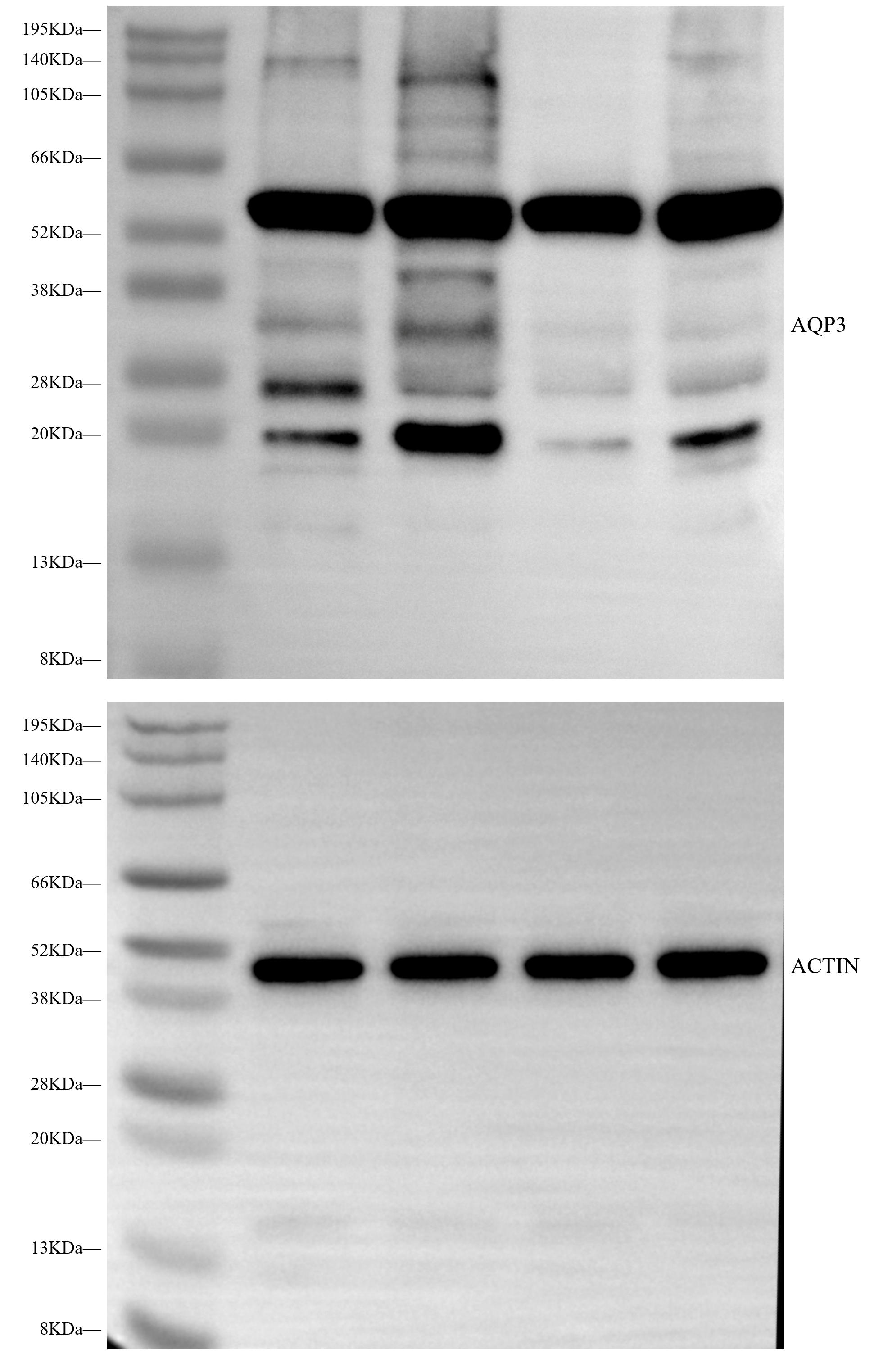

Supplement: Supplementary file 1 [file animals-15-00034-s001.zip › Original Images for Blots/三.tif]

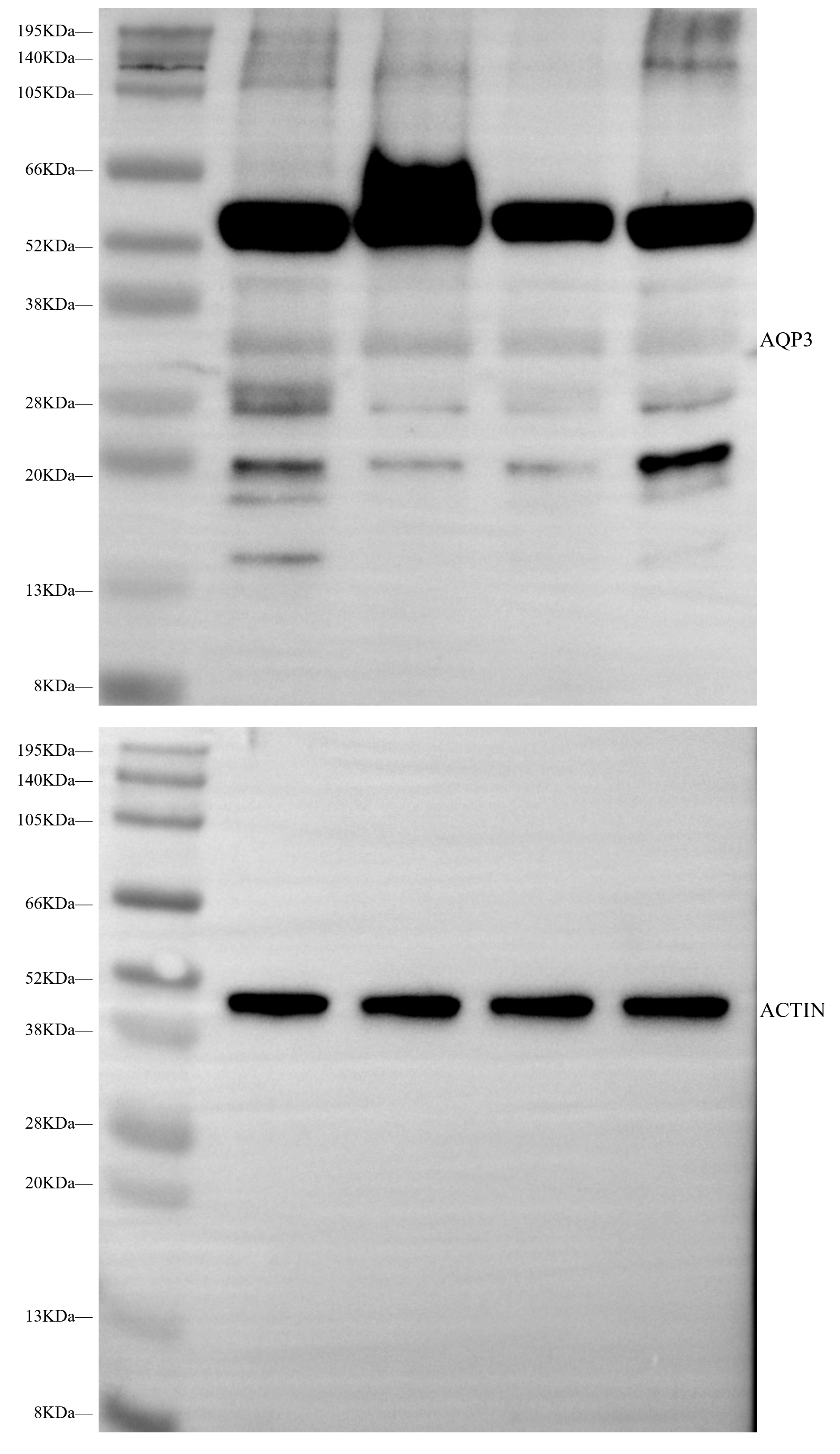

Supplement: Supplementary file 1 [file animals-15-00034-s001.zip › Original Images for Blots/二.tif]
